# Multiple origins of endosymbiosis within the Enterobacteriaceae (γ-Proteobacteria): convergence of complex phylogenetic approaches

**DOI:** 10.1186/1741-7007-9-87

**Published:** 2011-12-28

**Authors:** Filip Husník, Tomáš Chrudimský, Václav Hypša

**Affiliations:** 1 Faculty of Science, University of South Bohemia, Branišovská 31, České Budějovice 37005, Czech Republic; 2 Institute of Parasitology, Biology Centre of ASCR, Branišovská 31, České Budějovice 37005, Czech Republic

## Abstract

**Background:**

The bacterial family Enterobacteriaceae gave rise to a variety of symbiotic forms, from the loosely associated commensals, often designated as secondary (S) symbionts, to obligate mutualists, called primary (P) symbionts. Determination of the evolutionary processes behind this phenomenon has long been hampered by the unreliability of phylogenetic reconstructions within this group of bacteria. The main reasons have been the absence of sufficient data, the highly derived nature of the symbiont genomes and lack of appropriate phylogenetic methods. Due to the extremely aberrant nature of their DNA, the symbiotic lineages within Enterobacteriaceae form long branches and tend to cluster as a monophyletic group. This state of phylogenetic uncertainty is now improving with an increasing number of complete bacterial genomes and development of new methods. In this study, we address the monophyly versus polyphyly of enterobacterial symbionts by exploring a multigene matrix within a complex phylogenetic framework.

**Results:**

We assembled the richest taxon sampling of Enterobacteriaceae to date (50 taxa, 69 orthologous genes with no missing data) and analyzed both nucleic and amino acid data sets using several probabilistic methods. We particularly focused on the long-branch attraction-reducing methods, such as a nucleotide and amino acid data recoding and exclusion (including our new approach and slow-fast analysis), taxa exclusion and usage of complex evolutionary models, such as nonhomogeneous model and models accounting for site-specific features of protein evolution (CAT and CAT+GTR). Our data strongly suggest independent origins of four symbiotic clusters; the first is formed by *Hamiltonella *and *Regiella *(S-symbionts) placed as a sister clade to *Yersinia*, the second comprises *Arsenophonus *and *Riesia *(S- and P-symbionts) as a sister clade to *Proteus*, the third *Sodalis*, *Baumannia*, *Blochmannia *and *Wigglesworthia *(S- and P-symbionts) as a sister or paraphyletic clade to the *Pectobacterium *and *Dickeya *clade and, finally, *Buchnera *species and *Ishikawaella *(P-symbionts) clustering with the *Erwinia *and *Pantoea *clade.

**Conclusions:**

The results of this study confirm the efficiency of several artifact-reducing methods and strongly point towards the polyphyly of P-symbionts within Enterobacteriaceae. Interestingly, the model species of symbiotic bacteria research, *Buchnera *and *Wigglesworthia*, originated from closely related, but different, ancestors. The possible origins of intracellular symbiotic bacteria from gut-associated or pathogenic bacteria are suggested, as well as the role of facultative secondary symbionts as a source of bacteria that can gradually become obligate maternally transferred symbionts.

## Background

One of the most fundamental evolutionary questions concerning insect-bacteria symbiosis is the origin and phylogenetic relationships of various symbiotic lineages. This knowledge is necessary for understanding the dynamics and mechanisms of symbiosis establishment and maintenance within the host. For instance, close relationships between symbionts and pathogenic bacteria suggests a transition from pathogenicity to symbiosis; polyphyly of the symbionts within a single host group is evidence of their multiple independent origins and close relationships among symbionts of different biology indicate high ecological flexibility within a given symbiotic group [1-6]. These implications are particularly important within Enterobacteriaceae, the group containing a broad spectrum of symbiotic lineages and forms described from various groups of insects. Their biology varies from loosely associated facultative symbionts (often called Secondary (S) symbionts) to obligatory mutualists of a highly derived nature, called Primary (P) symbionts [7-9]. However, the concept of the P- and S-symbionts and the associated terminology are a major oversimplification and they become inadequate for the description of the ever increasing complexity of the symbiotic system within Enterobacteriaceae. This complexity is manifested by such phenomena as the presence of multiple symbionts in a single host [10], occurrence of intermediate symbiotic forms and the replacement of symbionts within a host [11-14] or close phylogenetic relationships between typical S- and P-symbionts revealing their high ecological versatility [15]. A good example of such a complex system is provided by the occurrence of multiple obligate symbionts within Auchenorrhyncha [10], universally harboring *Sulcia muelleri *(Bacteroidetes) [16] with either *Hodgkinia cicadicola *(α-Proteobacteria) in cicadas, *Zinderia insecticola *(β-Proteobacteria) in spittlebugs or *Baumannia cicadellinicola *(γ-Proteobacteria) in sharpshooters. All of these latter symbionts are obligate and have been cospeciating with their hosts for millions of years [17-21]. A close phylogenetic relationship between typical S- and P-symbionts has been so far demonstrated in two well defined and often studied groups, the enterobacterial genera *Arsenophonus *and *Sodalis *[5,22,23]. The general capability of S-symbionts to supplement the metabolic functions of P-symbionts or even replace them was demonstrated experimentally by replacement of *Buchnera *with *Serratia *in aphids [24].

It is obvious that all these fascinating processes can only be studied on a reliable phylogenetic background [9,25-28]. Unfortunately, under current conditions, the phylogeny within Enterobacteriaceae and the placement of various symbiotic lineages are very unstable. Particularly, the P-symbionts present an extremely difficult challenge to phylogenetic computation due to their strongly modified genomes [9]. There are several root problems that are responsible for this dissatisfactory state. Traditionally, 16S rDNA was frequently used as an exclusive molecular marker for the description of a new symbiont. Many lineages are thus represented only by this gene, which has been shown within Enterobacteriaceae to be inadequate for inferring a reliable phylogeny [29]. In addition, it is notoriously known that the phylogenetic information of symbiotic bacteria is often seriously distorted due to the conditions associated with the symbiotic lifestyle. The effect of strong bottlenecks accompanied by reduced purifying selection and the overall degeneration of symbiotic genomes have been thoroughly discussed in many studies [30-33]. As a result of these degenerative processes, symbiotic lineages may experience parallel changes of their DNAs and these convergences produce the main source of phylogenetic artifacts. Among the most important features are biased nucleotide composition favoring adenine-thymine bases and rapid sequence evolution. While the compositional bias leads to the introduction of homoplasies at both nucleotide and amino acid levels, the accelerated evolution is a well known source of the long-branch attraction phenomenon [34,35]. Due to these circumstances, symbionts almost always appear as long branches in phylogenetic trees and tend to cluster together [36].

Various methodological approaches have been tested to overcome these difficulties (Additional file 1). They are based mainly on the concatenation of a large number of genes through the whole genome [37-39], the supertree and the consensus approach [37], exclusion of amino acids (FYMINK: phenylalanine, tyrosine, methionine, isoleucine, asparagine and lysine) most affected by nucleotide bias [37], modifications of sequence evolution models [11,12,36,40] and use of the genome structure as a source of phylogenetic data [41]. Phylogenomic studies based on large concatenated sets frequently imply monophyly of the typical P-symbionts (Additional file 1). However, due to the limited number of available genomes, these studies are usually based on inadequate taxon sampling. For example, secondary symbionts and plant pathogens that were shown to break the P-symbiont monophyly in the analysis using a nonhomogeneous model [40] could not be included into these phylogenomic studies. It is important to note that P-symbionts are probably only distantly related to the *Escherichia*/*Salmonella*/*Yersinia *clade. Therefore, the monophyly of P-symbionts derived from such a phylogenomic dataset is logically inevitable, but does not carry any evolutionary information.

The non-monophyletic nature of P-symbionts has been recently suggested in several studies. Perhaps the most inspiring is a study based on a nonhomogeneous model that separates P-symbionts into two independent lineages [40]. As an alternative, a paraphyletic arrangement of these symbionts in respect to several free-living taxa has been revealed from gene-order analysis based on break-point and inversion distances [41]. Most recently, Williams *et al. *[42] performed a 'telescoping' multiprotein phylogenomic analysis of 104 γ-Proteobacterial genomes. The phylogeny of Enterobacteriaceae endosymbionts was difficult to resolve, although it appeared that there were independent origins of at least the *Sodalis *and *Buchnera *lineages.

Thus, there is now a spectrum of hypotheses on the phylogeny of insect symbionts, ranging from complete polyphyly with multiple independent origins to complete monophyly with one common origin. In this study, we take advantage of current progress in computational methods to investigate phylogenetic relationships among the symbiotic lineages. One of the promising recent methodological advances is the introduction of a site-heterogeneous non-parametric mixture CAT model that allows for site-specific features of protein evolution [43]. This model was shown to solve the long-branch attraction (LBA) artifacts and outperform the previous models [44-47]. Similarly, the slow-fast method based on removal of the fastest evolving sites was shown to reduce phylogenetic artifacts [48-54], as well as purine/pyrimidine (RY) data recoding [55-58] or amino acid data recoding [59,60]. We used these methods as the core of a complex approach and tried to investigate series of methods, models and parameters to detect common trends in changes of the topologies. To do this, we applied two parallel approaches, one based on the application of recently developed algorithms and the other on the removal or recoding of the positions most affected by rapid sequence evolution and/or compositional (AT) bias. In addition, we paid particular attention to the sampling and used as much of a complete set of both symbiotic and free-living lineages as possible. This approach is particularly important to avoid interpretation uncertainties due to the absence of phylogenetically important lineages.

## Results

**Figure 1:**
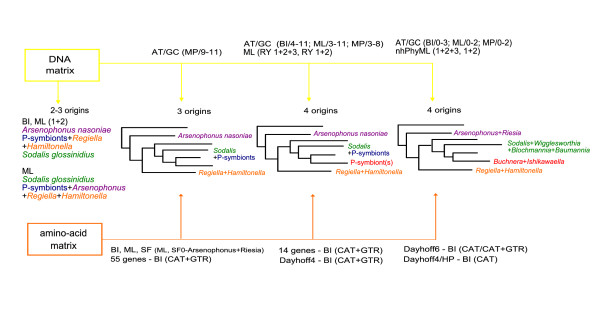
**Study design**. General design of the study summarizing all analyses and results. Individual topologies show the gradient of acquired results; method names are written above and below the arrows. Notice an increasing number of independent origins of symbionts and decreasing number of phylogenetic artifacts along the continuum towards the 'derived' methods. 1+2: third codon positions excluded; AT/GC(BI/4-11): AT/GC datasets 4-11 analyzed by BI; BI: Bayesian inference; Dayhoff6/Dayhoff4/HP: amino acid recoded matrices; ML: maximum likelihood; nhPhyML: ML under nonhomogeneous model; MP: maximum parsimony; RY: purine/pyrimidine recoded matrix; SF: slow-fasted datasets.

The complete methodological design of this study and the resulting topologies are depicted in Figure 1. All matrices, alignments and phylogenetic trees are available in the TreeBASE database http://purl.org/phylo/treebase/phylows/study/TB2:S11451, as supplementary material, or on the webpage http://users.prf.jcu.cz/husnif00.

### Standard maximum likelihood and Bayesian inference

**Figure 2:**
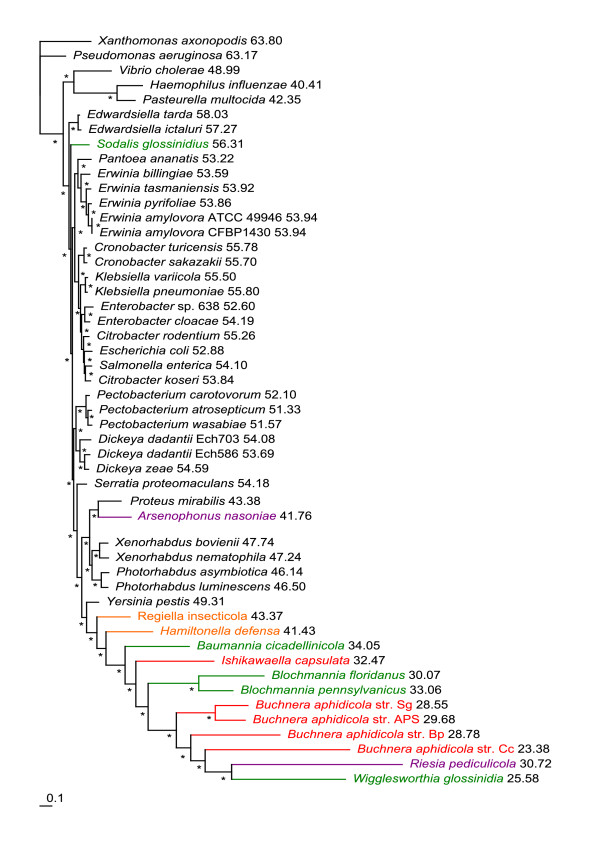
**MrBayes phylogram - 69 genes, nucleotide matrix**. Phylogenetic tree inferred from the concatenated nucleotide matrix using BI under the GTR+I+Γ model. Asterisks designate nodes with posterior probabilities equal to 1.0, values next to species names represent GC content calculated from the 69-gene dataset, genomic GC content can be found in Additional file 4. BI: Bayesian inference.

**Figure 3:**
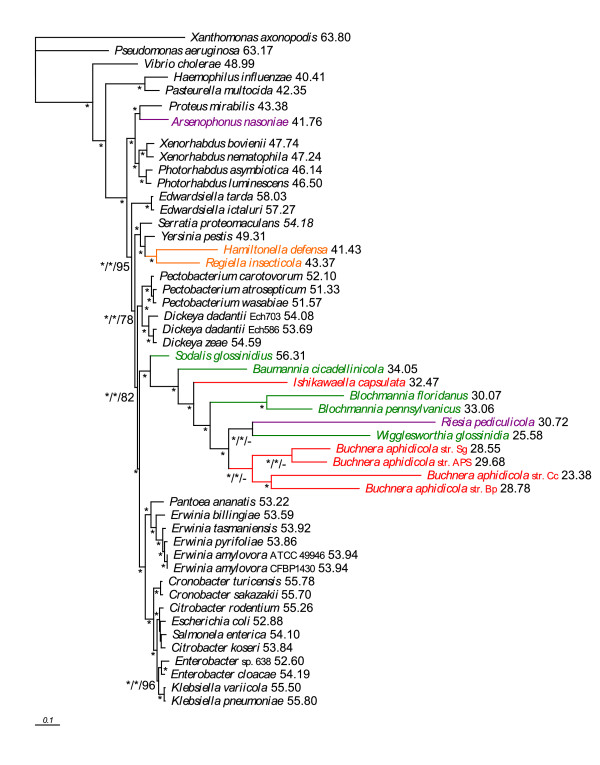
**MrBayes phylogram - 69 genes, amino acid matrix**. Phylogram inferred from the concatenated amino acid matrix using BI under the WAG+I+Γ model. Values at nodes represent posterior probabilities (WAG+I+Γ model, GTR+I+Γ protein model) and bootstrap supports from ML analysis (LG+I+Γ model). Asterisks designate nodes with posterior probabilities or bootstrap supports equal to 1.0, dashes designate values lower than 0.5 or 50, values next to species names represent GC content calculated from the 69-gene dataset, genomic GC content can be found in Additional file 4. BI: Bayesian inference. ML: maximum likelihood.

The single gene maximum likelihood (ML) analyses of both nucleic and amino acid data provided an array of mutually exclusive topologies. The majority consensus based on amino acid data (Additional file 2a) groups almost all symbionts into polytomy with only two pairs of sister symbiotic species being resolved (*Buchnera *and *Blochmannia*). Phylogenetic trees inferred by ML and Bayesian inference (BI) from the nucleic acid concatenated data using the General Time Reversible model with an estimated proportion of invariable sites (I) and heterogeneity of evolutionary rates modeled by the four substitution rate categories of the gamma (Γ) distribution with the gamma shape parameter (alpha) estimated from the data (GTR+I+Γ) were apparently affected by phylogenetic artifacts, as demonstrated by placement of *Riesia *and *Wigglesworthia *within the *Buchnera *cluster with high posterior probabilities in the BI tree (Figure 2) and the attraction of two outgroup species (*Haemophilus *and *Pasteurella*) in the ML tree with high bootstrap support (Additional file 2b). Similar topologies were also retrieved from the amino acid concatenate by ML and BI using the LG+I+Γ, WAG+I+Γ and GTR+I+Γ models (Figure 3). Nevertheless, in contrast to the nucleotide-derived results, the monophyly of the *Buchnera *clade was not disrupted and *Hamiltonella *and *Regiella *were unambiguously separated from the other symbionts and clustered with *Yersinia*.

### PhyloBayes, non-homogenous PhyML and modified matrices

**Figure 4:**
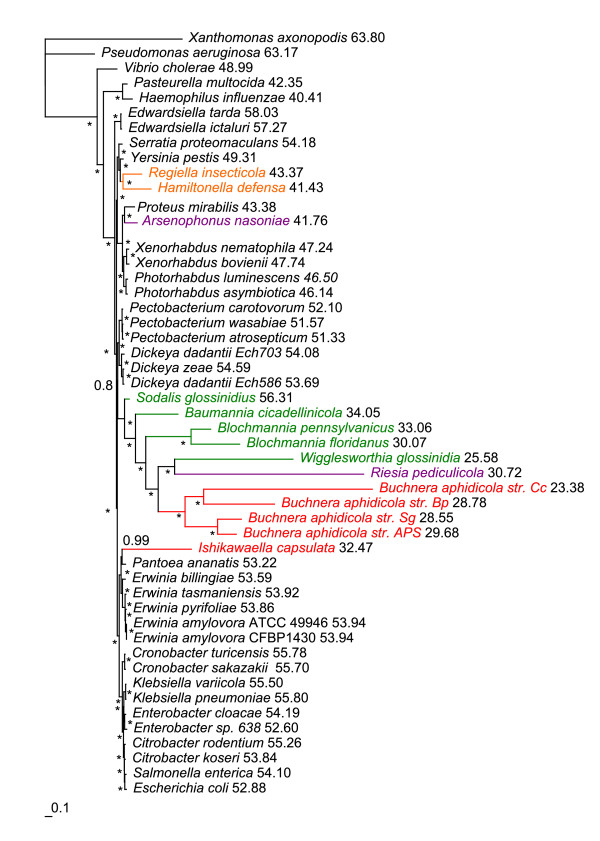
**PhyloBayes phylogram - 14 genes, amino acid matrix**. Phylogram derived from concatenation of 14 genes (*AceE*, *ArgS*, *AspS*, *EngA*, *GidA*, *GlyS*, *InfB*, *PheT*, *Pgi*, *Pnp*, *RpoB*, *RpoC*, *TrmE *and *YidC*) using PhyloBayes under the CAT+GTR model. Asterisks designate nodes with posterior probabilities equal to 1.0, values next to species names represent GC content calculated from the 69-gene dataset, genomic GC content can be found in Additional file 4.

The phylogenetic trees acquired under the CAT+GTR PhyloBayes model from 14 and 55 concatenated genes (Figure 4 and Additional file 2p) split symbiotic bacteria into four and three independent lineages, respectively. First, *Arsenophonus nasoniae *is a sister species to *Proteus mirabilis*; second, *Hamiltonella *and *Regiella *form a sister clade to *Yersinia pestis*; third, the *Sodalis*, *Baumannia*, *Blochmannia*, *Wigglesworthia*, *Riesia *and *Buchnera *clade form a sister clade to *Dickeya*/*Pectobacterium*. The position of *Ishikawaella *differs between the two datasets. In the 14-gene dataset, *Ishikawaella *forms a sister clade to *Pantoea *(Figure 4) and in the 55-gene dataset, it is attracted to the P-symbiont cluster (Additional file 2p).

**Figure 5:**
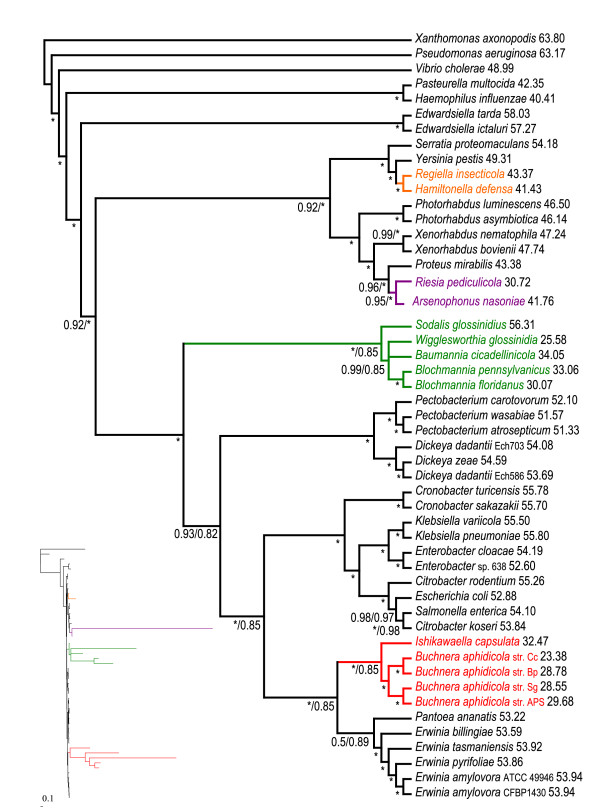
**PhyloBayes cladogram - 69 genes, Dayhoff6 amino acid recoded matrix**. Cladogram inferred from amino acid matrix recoded with Dayhoff6 scheme using PhyloBayes with the CAT and CAT+GTR model. Because of the length of symbiotic branches, phylogram is presented only as a preview (original phylogram can be found in Additional trees on our website). Values at nodes represent posterior probabilities from CAT and CAT+GTR analyses, respectively (asterisks designate nodes with posterior probabilities equal to 1.0). Values next to species names represent GC content calculated from the 69-gene dataset, genomic GC content can be found in Additional file 4.

A topology with four independent symbiotic clades resulted from the trees derived from dayhoff6 and dayhoff4 recoded amino acid data sets analyzed by CAT and CAT+GTR models (Figure 5, Additional file 2r, q) and partially with the hp (hydrophobic-polar) recoded dataset (Additional file 2c) - which was on the other hand affected by the substantial loss of phylogenetic information. The first clade is *Buchnera*+*Ishikawaella *as a sister clade to the *Erwinia*/*Pantoea *clade, the second clade is *Riesia*+*Arsenophonus *as a sister clade to *Proteus*, the third clade is *Hamiltonella*+*Regiella *as a sister clade to *Yersinia*, and the last clade is composed of *Sodalis*, *Baumannia*, *Blochmannia *and *Wigglesworthia*.

The analyses testing each symbiont independently, using a CAT+GTR model on the dayhoff6 recoded datasets, resulted in topologies supporting multiple origins of endosymbiosis (Additional file 2s). *Arsenophonus *clusters with *Proteus*; *Hamiltonella *clusters with *Yersinia*; *Regiella *clusters with *Yersinia*; and *Sodalis*, *Blochmannia*, *Baumannia*, *Riesia *and *Wigglesworthia *grouped into polytomy with the basal enterobacterial clades. Most importantly, the *Buchnera *clade clusters as a sister clade to the *Erwinia *clade and *Ishikawaella *is placed in polytomy with the *Pantoea *and *Erwinia *clade.

**Figure 6:**
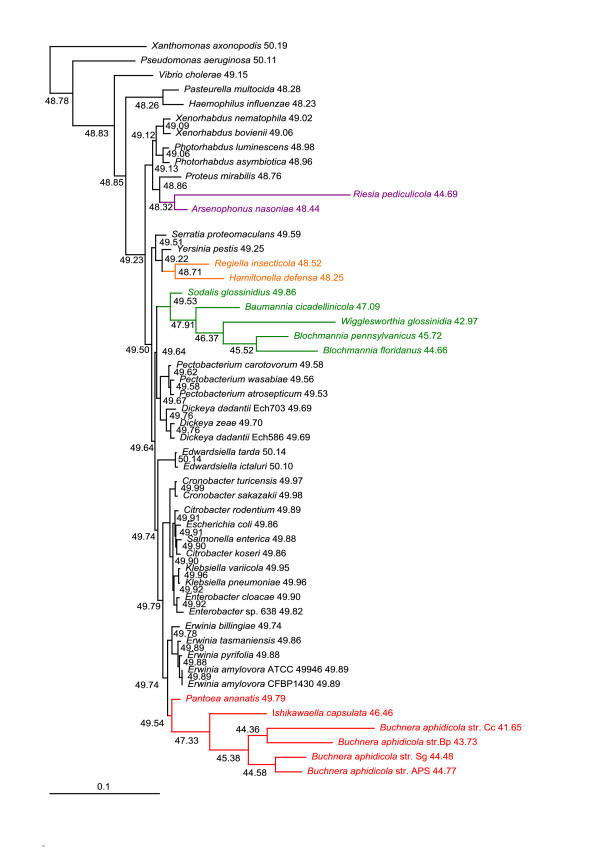
**nhPhyML phylogram - 69 genes, nucleotide matrix, third positions excluded**. Phylogram inferred from the concatenated nucleotide matrix without third codon positions using the nonhomogeneous model of evolution as implemented in nhPhyML. Values at nodes and branches represent GC content.

The non-homogenous (nh) PhyML nucleotide analyses with two different starting trees resulted in two different topologies (Figure 6 and Additional file 2d, e, f). When compared by the approximately unbiased (AU) test, the topology with four independent origins of symbiotic bacteria prevailed (*P *= 1) over the topology with monophyly of P-symbionts, which therefore corresponds to a local minimum due to a tree search failure (complete matrix: *P *= 2 × 10^-67^; matrix without the third positions: *P *= 9 × 10^-87^). The only incongruence in topologies based on the complete matrix (Additional file 2d) and the matrix without the third positions (Figure 6) was the placement of the *Sodalis*+*Baumannia*+*Blochmannia*+*Wigglesworthia *clade as a sister clade to the *Edwardsiella *or *Dickeya*/*Pectobacterium *clades.

Matrices obtained by removing positions according to the AT/GC contents produced trees covering the whole continuum illustrated in Figure 1. The most severe restrictions, that is, removal of all positions that contain both AT and GC categories or relaxing for up to three taxa (see BI trees in Additional file 2g, h, i, j), yielded topologies compatible with the results of the CAT model applied on the recoded amino acid data and of the nhPhyML analysis. Further relaxing the restriction rule led to a variety of trees along the Figure 1 continuum, with a less clear relation between the used parameter and the resulting topology (Additional file 3).

Compared to the ML analysis of all nucleotide positions, the analysis of first plus second positions reduced the obvious artifact of outgroup attraction (Additional file 2k). Nevertheless, it also sorted symbionts according to their branch length. Analysis of the RY recoded nucleotide matrix produced a tree compatible with the results of the CAT+GTR model (Additional file 2l). Analysis of the RY recoded nucleotide matrix without the third positions resulted in a topology with a *Sodalis+Baumannia+Blochmannia *cluster (as a sister to the *Pectobacterium*/*Dickeya *clade) separated from the rest of the P-symbionts, which clustered with the *Erwinia*/*Pantoea *clade (Additional file 2m). Slow-fast analyses with gradual reduction of saturated positions did not produce the polyphyly of P-symbionts (Additional file 3; only the first five trees presented, subsequent trees are identical to the fifth tree). However, this analysis shows an increasing effect of LBA artifacts associated with the increasing number of remaining saturated positions, especially *Riesia *attraction and swapping of symbiotic branches according to their length.

## Discussion

### Performance of the methods: convergence towards non-monophyly

The results obtained in this study strongly indicate that the frequently retrieved monophyly of P-symbionts is an artifact caused by their highly modified genomes. None of the most widely used methods, that is, ML and BI with different models used on nucleic (GTR+I+Γ) and amino acid (GTR/LG/WAG+I+Γ) data, were capable of resolving deep phylogenetic relationships and correct placement of the symbiotic taxa. This conclusion is evidenced by obvious artifacts, such as the inclusion of *Riesia *into the P-symbiotic lineage or the even more conspicuous distorted placing of *Wigglesworthia *within the *Buchnera *cluster. The arrangement of such trees suggests that these methods sort the symbionts according to their branch lengths and/or AT contents and attach the whole symbiotic cluster to the longest branch available. While the difficulty with placement of the most aberrant taxa, such as *Riesia*, *Wigglesworthia *and *Buchnera *(*Cinara cedri*) was also observed when using the mixture model accounting for site specific characteristics of protein evolution (Figure 4; Additional files 2p and 5), these artifacts disappeared after amino acid data recoding followed by CAT and CAT+GTR model analysis and the application of a nonhomogeneous model.

Additional support for the non-monophyly view stems from the second, parallel approach based on the restricted matrices. While our newly developed method shares the basic principles with the slow-fast and recoding methods, such as the removal of the positions that are likely to distort the phylogenetic relationships due to their aberrant evolution, it differs in the criteria of their removal and thus produces different input data. It is therefore significant that this method led independently to the same picture, the non-monophyly of the P-symbionts with clustering identical to the above analyses: *Ishikawaella*+*Buchnera *and *Sodalis*+*Baumannia*+*Blochmannia*+*Wigglesworthia*. The removal of the heteropecillous sites was recently shown to have similar effectiveness as our new method [61], which further supports the results. Moreover, this topology was obtained even under the maximum parsimony (MP) criterion (Additional file 3), which is known to be extremely sensitive to LBA [34]. On the other hand, although slow-fast analysis is generally considered a powerful tool for resolving relationships among taxa with different rates of evolution, we show in our data that the mere exclusion of the fast evolving sites is not sufficient when using empirical models and should be followed by analysis using some of the complex models, such as the CAT-like models. In addition, since this method usually requires an *a priori *definition of monophyletic groups, it should be used and interpreted with caution. Similar to the slow-fast method, RY recoding and exclusion of third codon positions were not sufficient for resolving deep symbiont phylogeny. However, all these methods can remove at least some of the artifacts and provide insight for further analyses.

Summarizing the topologies obtained in this study (Figure 1), a convergence can be detected towards a particular non-monophyletic arrangement of P-symbionts, as revealed under the most 'derived' methods. This result strongly supports the view of multiple origins of insect endosymbionts, as first revealed by the nonhomogeneous model of sequence evolution [40], and is partially congruent with the analyses of gene order [41] and phylogenomics of Gammaproteobacteria [42]. It is also important to note that, apart from multiple symbiont clustering, the arrangement of the non-symbiotic taxa corresponds to most of the phylogenomic analyses using *Escherichia*/*Salmonella*/*Yersinia *taxon sampling [37-39].

### Biological significance of P-symbionts non-monophyly

Considering that most of the 'artifact-resistant' analyses point towards the non-monophyly of enterobacterial P-symbionts, the questions of how many symbiotic lineages are represented by the known symbiotic diversity and what are their closest free-living relatives now becomes of particular importance. It is not clear whether the split of the original P-symbiotic cluster into two lineages is definite or these two groups will be further divided after yet more sensitive methods and more complete data are available. At the moment there are still several clusters composed exclusively of derived symbiotic forms. In principle, three different processes may be responsible for the occurrence of such clusters: first, horizontal transmission of established symbiotic forms among host species; second, inadequate sampling with missing free-living relatives; or third, phylogenetic artifacts. All of these factors are likely to play a role in the current topological patterns. Being the main issues of this study, the role of methodological artifacts has been discussed above. Horizontal transmission, as the basis of non-artificial symbiotic clusters, is likely to take part at least in some cases. Perhaps the most convincing example is the *Wolbachia *cluster [62]: while within Enterobacteriaceae it may apply to *Arsenophonus*, *Sodalis *and possibly some other S-symbionts.

Recognition of the third cause, the incomplete sampling, and identification of the closest free-living relatives, now becomes a crucial step in future research. It is often assumed that symbionts originate from bacteria common to the environment typical for a given insect group. For example, cicadas spend most of their life cycle underground and feed primarily on plant roots. Consequently, their α-Proteobacterial symbiont *Hodgkinia cicadicola *originated within Rhizobiales [19]. A similar ecological background can be noticed in yet different hosts, the ixodid and argasid ticks. Several reports have shown that some of the tick-transmitted pathogens are related to their symbiotic fauna [63-65]. Many of the insect taxa associated with symbiotic Enterobacteriaceae are phytophagous, and plant pathogens thus fit well into this hypothesis as hypothetical ancestors of various insect symbionts lineages. The presence of a type III secretion system, which is used in pathogenic bacteria for host cell invasion, in secondary symbionts [66-69] and its remnant in the primary symbiont of *Sitophilus *spp. weevils [70] could further support the theory of pathogenic ancestors of insect symbionts. It can only be speculated that these bacteria first became S-symbiont type and were horizontally transferred to various other insect species. Within some of the infected species, facultative symbionts eventually became obligatory primary symbionts. An identical situation can be observed in symbiotic clades with numerous species, such as *Wolbachia *[71,72], *Sodalis *[23,73,74] or *Arsenophonus *[5].

In our study, we gave particular attention to the sampling of free-living Enterobacteriaceae to provide as complete a background for the symbiotic lineages as possible under the current state of knowledge (that is, the availability of the genomic data). The most consistent picture derived from the presented analyses places the four main symbiotic clusters into the following positions. First, for the *Buchnera *cluster, its previously suggested relationship to *Erwinia *was confirmed. *Erwinia*, as a genus of mostly plant pathogenic bacteria, has been previously suggested to represent an ancestral organism, which upon ingestion by aphids at least 180 million years ago [75] turned into an intracellular symbiotic bacterium [76]. However, it is not known whether it was primarily pathogenic to aphids, similar to *Erwinia aphidicola *[77], or a gut associated symbiotic bacterium as in pentatomid stinkbugs [78], thrips [79,80] or Tephritidae flies [81-83]. *Ishikawaella capsulata*, an extracellular gut symbiont of plataspid stinkbugs [84], was the only symbiotic bacterium that clustered in our 'derived' analyses with the *Buchnera *clade. However, several single-gene studies indicate that this group contains some additional symbiotic lineages for which sequenced genome data is not currently available. These are, in particular, the extracellular symbionts of acanthosomatid stinkbugs [85], parastrachid stinkbugs [86], scutellerid stinkbugs [87,88] and some of the symbionts in pentatomid stinkbugs [78].

The second clade, represented in our analysis by *Sodalis+Baumannia+Blochmannia+Wigglesworthia*, is likely to encompass many other P- and S-symbionts [89-92]. The possible single origin of these symbionts has to be further tested, however the interspersion of both forms, together with basal position of *Sodalis*, seem to support a transition from a secondary to primary symbiotic lifestyle [15]. In our analysis, the whole clade was placed between pathogenic bacteria of plants and animals, the *Edwardsiella *and *Pectobacterium*/*Dickeya *clades, or as a sister to the latter group. Recently, another symbiotic bacterium (called BEV, *Euscelidius variegatus *host) was shown to be a sister species to *Pectobacterium *[93].

Two additional independent origins of insect symbionts are represented by the *Arsenophonus*/*Riesia *clade and *Hamiltonella*+*Regiella*. Both of these clades clustered in our analyses in the positions indicated by previous studies, that is, as related to *Proteus *and *Yersinia*, respectively [5,67,93-97].

While the position of individual symbiotic lineages is remarkably consistent across our 'artifact-resistant' analyses and are well compatible with some of the previous studies, the topology can only provide a rough picture of the relationships within Enterobacteriaceae. To get a more precise and phylogenetically meaningful background for an evolutionary interpretation, the sample of free-living bacteria as a possible source of symbiotic lineages has to be much improved. An illuminating example is provided by the bacterium *Biostraticola tofi*, described from water biofilms. When analyzed using 16S rDNA, this bacterium seemed to be closely related to *Sodalis *[98]. Its position as a sister group to the *Sodalis*/*Baumannia*/*Blochmannia*/*Wigglesworthia *clade was also retrieved in our single-gene analysis (*groEL*, data not shown). If confirmed by more precise multigene approach, *Biostraticola *would represent the closest bacterium to the large symbiotic cluster.

## Conclusions

The topologies obtained by several independent approaches strongly support the non-monophyletic view of enterobacterial P-symbionts. Particularly, they show that at least three independent origins led to highly specialized symbiotic forms, the first giving rise to *Sodalis*, *Baumannia*, *Blochmannia *and *Wigglesworthia *(S- and P-symbionts), the second to *Buchnera *and *Ishikawella *and the last to *Riesia *and *Arsenophonus *(S- and P-symbionts). This separation of symbiotic clusters poses an interesting question as to whether the presented disbandment of the P-symbiotic cluster is definite or if it will continue after yet more complete data are available and more realistic evolutionary models [99-101] are applied. One obvious drawback of the current state is that many additional symbiotic lineages already known within Enterobacteriaceae cannot be at the moment included into serious phylogenetic analyses due to the lack of sufficient molecular data and will have to be revisited once complete genomic data are available. These bacteria include symbionts of mealybugs [89,102], psyllids [90,103], lice [2,91], weevils [11,12,92], reed beetles [104,105], true bugs [78,84-88,106,107] and symbionts of leeches [108,109]. Similarly, the importance of free-living bacteria and variety of S-symbionts as possible ancestors of P-symbionts should not be underestimated when assembling datasets for phylogenetic analyses. The shift from polymerase chain reaction-based gene-centered sequencing towards high-throughput next-generation sequencing may soon provide sufficient data for more complete analyses of the Enterobacteriaceae phylogeny.

## Methods

### Matrices and multiple sequence alignments

The genes used in this study were extracted from 50 complete genome sequences of γ-Proteobacteria available in GenBank (Additional file 4), including 14 endosymbiotic Enterobacteriaceae. We did not include *Carsonella ruddii *[110] since this psyllid symbiotic bacterium does not appear to be a member of the Enterobacteriaceae clade [90,111] and is only attracted there by the AT rich taxa. After removal of the AT rich lineages from the analysis, *Carsonella ruddii *clusters with the genus *Pseudomonas *[42]. Also, we did not include *Serratia symbiotica *[95] because its genome only became available after completion of our datasets. However, the phylogenetic position of this symbiotic bacterium within *Serratia *genus is robust and was confirmed in several studies [6,14,112].

To minimize the introduction of a false phylogenetic signal, we compared the genomes of all symbiotic bacteria and selected only single-copy genes present in all of the included symbiotic and free-living taxa. Such strict gene exclusion was also necessary regarding the usage of computationally demanding methods; it was one of our goals to produce a taxonomically representative data set of efficient size with no missing data. Altogether, 69 orthologous genes, mostly involved in translation, ribosomal structure and biogenesis (Additional file 4) were selected according to the Clusters of Orthologous Groups of proteins (COGs) [113,114]. Single-gene nucleotide data sets were downloaded via their COG numbers from a freely available database (MicrobesOnline [115]).

All protein coding sequences were translated into amino acids in SeaView version 4 [116], aligned by the MAFFT version 6 L-INS-i algorithm [117] and toggled back to the nucleotide sequences. Ambiguously aligned positions (codons) were excluded by Gblocks v0.91b [118,119] with the following parameters: minimum number of sequences for a conserved position: 26; minimum number of sequences for a flanking position: 43; maximum number of contiguous nonconserved positions: 8; minimum length of a block: 10; allowed gap positions: with half. The resulting trimmed alignments were checked and manually corrected in BioEdit v7.0.5 [120]. Alignments were concatenated in SeaView. The 69 gene concatenate resulted in an alignment of 63, 462 nucleic acid positions with 42, 481 parsimony-informative and 48, 527 variable sites and 21, 154 amino acid positions with 12, 735 parsimony-informative and 15, 986 variable sites.

### Phylogenetic analyses

We used two different approaches to deal with the distortions caused by the highly modified nature of symbiotic genomes, which are the main source of the phylogenetic artifacts in phylogenetic analyses.

First, we applied complex models of molecular evolution. Using PhyloBayes 3.2f [121], we applied non-parametric site heterogeneous CAT and CAT+GTR models [43]. For all PhyloBayes analyses, we ran two chains with an automatic stopping option set to end the chain when all discrepancies were lower than 0.3 and all effective sizes were larger than 100. Under the CAT and CAT+GTR models, the four independent PhyloBayes runs were stuck in a local maximum (maxdiff = 1) even after 25, 000 and 10, 000 cycles, respectively, and we were not able to reach Markov Chain Monte Carlo (MCMC) convergence. Therefore, we present these trees only as supplementary material (although they mostly point toward multiple origins of symbiosis; Additional file 5) and we ran the CAT+GTR analyses with the reduced dataset based on 14 genes with the number of parsimony-informative amino acid positions higher than 300 (*AceE*, *ArgS*, *AspS*, *EngA*, *GidA*, *GlyS*, *InfB*, *PheT*, *Pgi*, *Pnp*, *RpoB*, *RpoC*, *TrmE *and *YidC*). To check for compatibility of these arbitrary selected 14 genes with the rest of the data, we also analyzed, in a separate analysis, the remaining 55-gene dataset under the CAT+GTR model. Using nhPhyML [122], we applied a nonhomogeneous nonstationary model of sequence evolution [123,124], which can deal with artifacts caused by compositional heterogeneity [40,125,126]. We used two different starting trees (Additional file 2n) and ran the analyses with and without the third codon positions. The resulting trees were evaluated by an AU test in CONSEL [127].

The second approach relies on the selective restriction of the data matrix. We used four previously established methods of data weighting and/or exclusion (see Background): RY data recoding, amino acid data recoding, exclusion of third codon positions and slow-fast analysis, and developed one additional method: since transition from G/C to A/T at many positions is a common homoplasy of symbiotic genomes, we removed from the matrix all positions containing both the G/C and A/T states. All substitutions considered in the subsequent analyses thus included exclusively transversions within the A/T or G/C categories. To analyze an effect of this restriction on the reduction of the data, we prepared 11 matrices with a partially relaxed rule (removing all positions with AT+GC, allowing for one taxon exception, two taxa exception, and so on, up until a 10 taxa exception). Since this method has never been tested, we analyzed the restricted matrices by the BI, ML (parameters as for standard analyses) and MP using PAUP* 4.0b10 with the tree bisection and reconnection algorithm [128]. Four other types of data weighting and/or exclusion were used to increase the phylogenetic signal to noise ratio and determine the robustness of our results. First, the third codon positions were removed in SeaView. Second, RY recoding was performed on all and first plus second positions. Third, saturated positions were excluded from the concatenated data sets by SlowFaster [129]. To assign substitutional rates to individual positions, unambiguously monophyletic groups were chosen on a polytomic tree (Additional file 2o), positions with the highest rates were gradually excluded and 21 restricted matrices were produced. These weighted data sets were analyzed by ML. Fourth, amino acid data recoding was performed in PhyloBayes with hp (A, C, F, G, I, L, M, V, W) (D, E, H, K, N, P, Q, R, S, T, Y), dayhoff4 (A, G, P, S, T) (D, E, N, Q) (H, K, R) (F, Y, W, I, L, M, V) (C = ?) and dayhoff6 (A, G, P, S, T) (D, E, N, Q) (H, K, R) (F, Y, W) (I, L, M, V) (C) recoding schemes. In addition, we have prepared 10 dayhoff6 recoded matrices to test individual symbiotic lineages without the presence of other symbionts. Amino acid recoded matrices were analyzed using the CAT and CAT+GTR models, which are more immune to phylogenetic artifacts than one-matrix models.

To allow for comparison of the results with previously published studies, as well as to separate the effect of newly used models and methods from changes due to the extended sampling, we also used standard procedures of phylogenetic inference, ML and BI. The following programs, algorithms and parameters were used in the ML and BI analyses. ML was applied to single-gene and concatenated alignments of both nucleotides and amino acids using PhyML v3.0 [130] with the subtree pruning and regrafting tree search algorithm. BI was performed in MrBayes 3.1.2 [131] with one to five million generations and tree sampling every 100 generations. Exploration of MCMC convergence and burn-in determination was performed in AWTY and Tracer v1.5 [132,133]. Evolutionary substitution models for proteins were selected by ProtTest 2.4 [134] and for DNA by jModelTest 0.1.1 [135] according to the Akaike Information Criterion. For DNA sequences, the GTR+I+Γ model was used [136-138]. Transition and transversion models [139] were used with I+Γ under ML for the first two AT/GC datasets. LG+I+Γ [140], WAG+I+Γ [141] and GTR+I+Γ models were used for amino acid data. A cross-validation method implemented in PhyloBayes [121,142] was used to estimate the fit of CAT-like models. For both datasets, the 14 selected genes as well as the complete 69 genes set, the cross-validation was performed according to the PhyloBayes manual in 10 replicates each with 1, 100 cycles. The CAT-Poisson model had significantly better fit to the data than the GTR model (Δ*l *157.37 ± 56.9379 for the 14-gene matrix and Δ*l *3923.9 ± 1963.5 for the 69-gene matrix); of the CAT-like models, the CAT+GTR model was found to be significantly better than the CAT-Poisson model (Δ*l *536.71 ± 32.8341 for the 14-gene matrix and Δ*l *1633.4 ± 123.482 for the 69-gene matrix) in all 10 replicates.

## Competing interests

The authors declare that they have no competing interests.

## Authors' contributions

FH carried out the sequence alignments and phylogenetic analyses, and participated in the study design, evolutionary interpretation of the results and preparation of the manuscript. TCH compiled and analyzed the AT/GC reduced matrices. VH conceived of the study and participated in its design, evolutionary interpretation of the results and preparation of the manuscript. All authors read and approved the final manuscript.

## Supplementary Material

Additional file 1 **Summary of 20 studies on symbionts phylogeny**. Click here for file

Additional file 2 **Additional phylogenetic trees**. Click here for file

Additional file 3 **All phylogenetic trees derived from AT-GC and SF datasets**. A rar file of all phylogenetic trees obtained under BI, ML and MP from 11 AT/GC datasets, and under ML from five slow-fasted datasets. Trees are in phylip and nexus formats and can be viewed, for example, in TreeView http://taxonomy.zoology.gla.ac.uk/rod/treeview.html or Mesquite http://mesquiteproject.org/mesquite/mesquite.html. Click here for file

Additional file 4 **List of the taxa and orthologous genes used in the study**. Click here for file

Additional file 5 **Additional phylogenetic trees inferred from CAT and CAT+GTR unconverged chains**. Click here for file
