# Supplementary material for: Multiple origins of endosymbiosis within the Enterobacteriaceae (γ-Proteobacteria): convergence of complex phylogenetic approaches
Source: BMC Biol. 2011 Dec 28;9:87. doi: 10.1186/1741-7007-9-87 (PMC3271043; doi:10.1186/1741-7007-9-87)
Supplement: Additional file 1 — Summary of 20 studies on symbionts phylogeny. [file 1741-7007-9-87-S1.DOC]

| Data | No. symb. | Symbiotic clusters | Method | Reference |
| --- | --- | --- | --- | --- |
| Single gene (16S rRNA) | 19 | *Buch+Sod+Wig+Blo* | Least-square distance, NJ, ML | [1] |
| Single gene (16S rRNA) | 9(+4) | *Ars (incl. Phlomobacter)*  *Sod*+*SOPE*+*Wig*+*Blo*  *Ham (Bemisia* S-symbiont)*+Anomoneura* Y-symbiont*+Buch* | nhML | [2] |
|  |  | *Buch*+*Wig*+*Blo*+*Anomoneura* Y-symb  *Ars+Sod*+*SOPE*+*Bemisia* S-symb | MP, ML, NJ |  |
| Multiple genes (61 genes concatenated) | 5 | *Blo*+*Wig*+*Buch* | BI, ML | [3] |
| Single gene (16S rRNA) | 2 | *Buch*+*Wig* | ML, NJ | [4] |
|  |  | *Buch*  *Wig* | nhNJ |  |
| Multiple genes (205 genes concatenated/consensus) |  | *Buch*+*Wig* | ML, NJ |  |
| Multiple genes (258 genes) | 3 | *Buch*+*Wig* | nhNJ, ML | [5] |
| Gene order (BP and INV distances)  Multiple genes (10 genes) | 5 | *Blo*  *Buch*+*Wig* | FM, NJ | [6] |
|  |  | *Blo*+*Wig*+*Buch* | ML |  |
| Single gene (16S rRNA) | 19 | *Por*  *Ham* (aphid T-type symbiont)  *Buch*+*Blo*+*Wig*+*Sod*+*SOPE*+symbionts of mealybugs and psyllids | BI, MP | [7] |
|  |  | *Por*  *Ham* (aphid T-type symbiont)  *Buch*  *Blo*+*Wig*+*Sod*+*SOPE*+symbionts of mealybugs and psyllids | ML |  |
|  |  | *Por*  *Ham* (aphid T-type symbiont)+*Buch*  *Blo*+*Wig*+*Sod*+*SOPE*+symbionts of mealybugs and psyllids | nhNJ |  |
| Single gene (*groEL*) | 9 | *Buch*  *Wig*+*Blo*+*Sod*+*SOPE*  *Por* | BI, nhNJ, nhML |  |
|  |  | *Buch+ Por+Wig*+*Blo*+*Sod*+*SOPE* | ML, MP |  |
| Multiple genes (31 genes) | 5 | *Blo*+*Wig*+*Buch* | ML | [8] |
| Multiple genes (61 genes) | 5 | *Blo*+*Wig*+*Buch* | nhML | [9] |
| Overlapping genes distance  (BPhyOG) | 2 | *Buch*  *Wig* | NJ, UPGMA | [10] |
| Multiple genes (45 genes) | 6 | *Blo*+*Wig*+*Buch*+*Bau* | ML | [11] |
| Single gene (16S rRNA) | 5 | *Blo*+*Wig*+*Buch* | MP, ML, LogDetNJ | [12] |
|  |  | *Blo+Wig*  *Buch* | nhNJ |  |
| Multiple genes (200 genes) concatenated/FYMINK removed/consensus/supertree |  | *Blo*+*Wig*+*Buch* | ML, MP, BI |  |
| Multiple genes (379 genes)  supertree |  |  | MRP, dfit, qfit, sfit |  |
| Multiple genes (579 genes)  concatenated/supertree |  |  | ML |  |
| Multiple genes (133, 200, 579 genes) concatenated/supertree | 5 | *Blo*+*Wig*+*Buch* | ML, MRP | [13] |
| Genome context networks | 4 | *Blo*+*Wig*+*Buch* | NJ | [14] |
| Overlapping genes distance (OGtree) | 2 | *Wig*+*Buch* | UPGMA, NJ, FM | [15] |
| Multiple genes (31 genes) | 9 | *Sod*+*Bau*+*Blo*+*Wig*+*Buch* | ML | [16] |
| Multiple genes (13, 36 genes) | 4 | *Wig*+*Buch*+*Bau*+*Sod* | NJ, ML, MP | [17] |
| Single gene (16S rRNA) | 7 | *Sulcia*+*Blattabacterium*  *Sod*+*Blo*+*Bau*+*Wig*+*Buch* | ML | [18] |
| COG categories–functional closeness (Kulczynski distance) | 7 | *Buch* str. Cc+*Sulcia*  *Sod*  *Wig*+*Buch* str. APS+*Bau*+*Blattabacterium*+*Blo* | Linkage clustering method |  |
| Single gene (16S rRNA)  Overlapping genes distance (OGtree2) | 5 | *Blo*+*Wig*+*Buch* | UPGMA, NJ, FM | [19] |
| Single gene (16S rRNA) | 9 | *Buch*+*Wig*+*Blo*+*Bau*+*Sod* | BI | [20] |
| Single gene (23S rRNA)  Multiple genes (16S+23S rRNA) | 5 | *Sod*  *Buch*+*Wig*+*Blo*+*Bau* | ML |  |
| Multiple genes (356-1,262 genes) |  | *Sod*  *Buch* |  |  |

Additional file 1 - Summary of 20 phylogenetic studies analysing positions of insect symbionts within Enterobacteriaceae. No. symb.=number of gammaproteobacterial symbionts used for the analysis. Methods acronyms: NJ=neighbor joining, ML=maximum likelihood, MP=maximum parsimony, BI=Bayesian inference, nhNJ/nhML=nonhomogeneous NJ/ML, FM=Fitch-Margoliash method, UPGMA=unweighted pair group method with arithmetic mean, MRP=matrix representation using parsimony, dfit=most similar supertree, qfit=maximum quartet fit, sfit=maximum splits fit, BP=break point distance, INV=inversion distance. Taxa abbreviations: Buch=*Buchnera*, Blo=*Blochmannia*, Wig=*Wigglesworthia*, Sod=*Sodalis*, Bau=*Baumannia*, Ham=*Hamiltonella*, Por=*Portiera* (*Bemisia tabaci* endosymbiont), Ars=*Arsenophonus*, SOPE=*Sitophilus oryzae* endosymbiont.

References

1. Sauer C, Stackebrandt E, Gadau J, Holldobler B, Gross R: **Systematic relationships and cospeciation of bacterial endosymbionts and their carpenter ant host species: proposal of the new taxon *Candidatus* Blochmannia gen. nov**. *Int J Syst Evol Microbiol* 2000, **50 Pt 5**:1877-1886.

2. Charles H, Heddi A, Rahbe Y: **A putative insect intracellular endosymbiont stem clade, within the Enterobacteriaceae, infered from phylogenetic analysis based on a heterogeneous model of DNA evolution**. *C R Acad Sci Ser III Sci Vie* 2001, **324**(5):489-494.

3. Gil R, Silva FJ, Zientz E, Delmotte F, Gonzalez-Candelas F, Latorre A, Rausell C, Kamerbeek J, Gadau J, Holldobler B *et al*: **The genome sequence of *Blochmannia floridanus*: comparative analysis of reduced genomes**. *Proc Natl Acad Sci U S A* 2003, **100**(16):9388-9393.

4. Lerat E, Daubin V, Moran NA: **From gene trees to organismal phylogeny in prokaryotes: the case of the γ-Proteobacteria**. *PLoS Biol* 2003, **1**(1):e19.

5. Canback B, Tamas I, Andersson SG: **A phylogenomic study of endosymbiotic bacteria**. *Mol Biol Evol* 2004, **21**(6):1110-1122.

6. Belda E, Moya A, Silva FJ: **Genome rearrangement distances and gene order phylogeny in γ-Proteobacteria**. *Mol Biol Evol* 2005, **22**(6):1456-1467.

7. Herbeck JT, Degnan PH, Wernegreen JJ: **Nonhomogeneous model of sequence evolution indicates independent origins of primary endosymbionts within the Enterobacteriales (γ-Proteobacteria)**. *Mol Biol Evol* 2005, **22**(3):520-532.

8. Ciccarelli FD, Doerks T, von Mering C, Creevey CJ, Snel B, Bork P: **Toward automatic reconstruction of a highly resolved tree of life**. *Science* 2006, **311**(5765):1283-1287.

9. Delmotte F, Rispe C, Schaber J, Silva FJ, Moya A: **Tempo and mode of early gene loss in endosymbiotic bacteria from insects**. *BMC Evol Biol* 2006, **6**:56.

10. Luo YQ, Fu C, Zhang DY, Lin K: **Overlapping genes as rare genomic markers: the phylogeny of gamma-Proteobacteria as a case study**. *Trends Genet* 2006, **22**(11):593-596.

11. Wu D, Daugherty SC, Van Aken SE, Pai GH, Watkins KL, Khouri H, Tallon LJ, Zaborsky JM, Dunbar HE, Tran PL *et al*: **Metabolic complementarity and genomics of the dual bacterial symbiosis of sharpshooters**. *PLoS Biol* 2006, **4**(6):e188.

12. Comas I, Moya A, Gonzalez-Candelas F: **From phylogenetics to phylogenomics: the evolutionary relationships of insect endosymbiotic γ-Proteobacteria as a test case**. *Syst Biol* 2007, **56**(1):1-16.

13. Comas I, Moya A, Gonzalez-Candelas F: **Phylogenetic signal and functional categories in Proteobacteria genomes**. *BMC Evol Biol* 2007, **7 Suppl 1**(S7).

14. Ding GH, Yu ZH, Zhao J, Wang Z, Li Y, Xing XB, Wang CA, Liu L, Li YX: **Tree of Life Based on Genome Context Networks**. *Plos One* 2008, **3**(10):e3357.

15. Jiang LW, Lin KL, Lu CL: **OGtree: a tool for creating genome trees of prokaryotes based on overlapping genes**. *Nucleic Acids Res* 2008, **36**:W475-W480.

16. Wu M, Eisen JA: **A simple, fast, and accurate method of phylogenomic inference**. *Genome Biol* 2008, **9**(10):R151.

17. Gao B, Mohan R, Gupta RS: **Phylogenomics and protein signatures elucidating the evolutionary relationships among the *Gammaproteobacteria***. *Int J Syst Evol Microbiol* 2009, **59**:234-247.

18. Lopez-Sanchez MJ, Neef A, Pereto J, Patino-Navarrete R, Pignatelli M, Latorre A, Moya A: **Evolutionary convergence and nitrogen metabolism in *Blattabacterium* strain Bge, primary endosymbiont of the cockroach *Blattella germanica***. *Plos Genet* 2009, **5**(11):e1000721.

19. Cheng CH, Yang CH, Chiu HT, Lu CL: **Reconstructing genome trees of prokaryotes using overlapping genes**. *BMC Bioinformatics* 2010, **11**:102.

20. Williams KP, Gillespie JJ, Sobral BW, Nordberg EK, Snyder EE, Shallom JM, Dickerman AW: **Phylogeny of Gammaproteobacteria**. *J Bacteriol* 2010, **192**(9):2305-2314.
