# Supplementary material for: Multiple origins of endosymbiosis within the Enterobacteriaceae (γ-Proteobacteria): convergence of complex phylogenetic approaches
Source: BMC Biol. 2011 Dec 28;9:87. doi: 10.1186/1741-7007-9-87 (PMC3271043; doi:10.1186/1741-7007-9-87)
Supplement: Additional file 2 — Additional phylogenetic trees. [file 1741-7007-9-87-S2.DOC]

Additional file 2 - Additional phylogenetic trees.


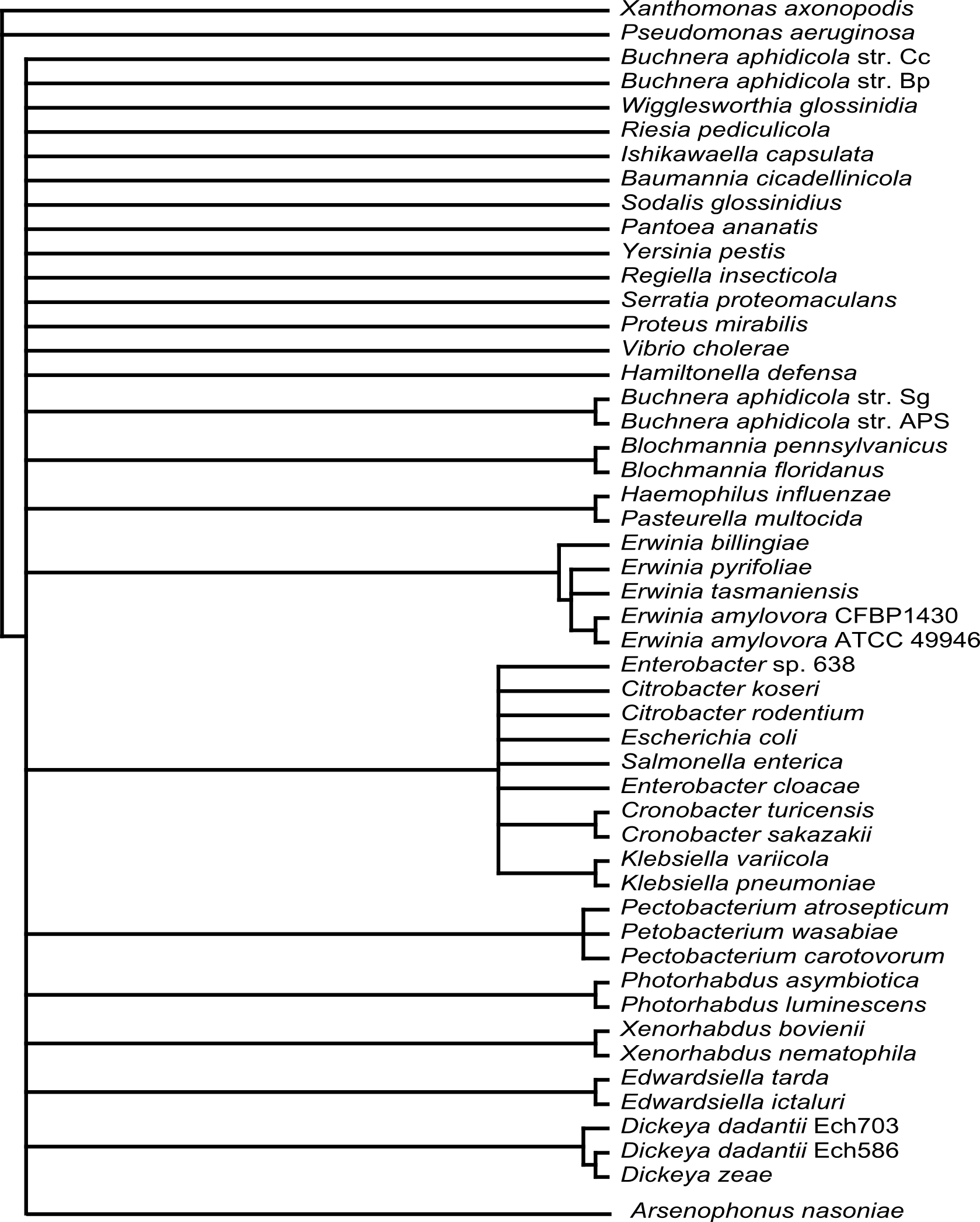


Additional file 2a - Majority-rule consensus inferred from 69 amino-acid gene trees using PAUP* 4.0b10.


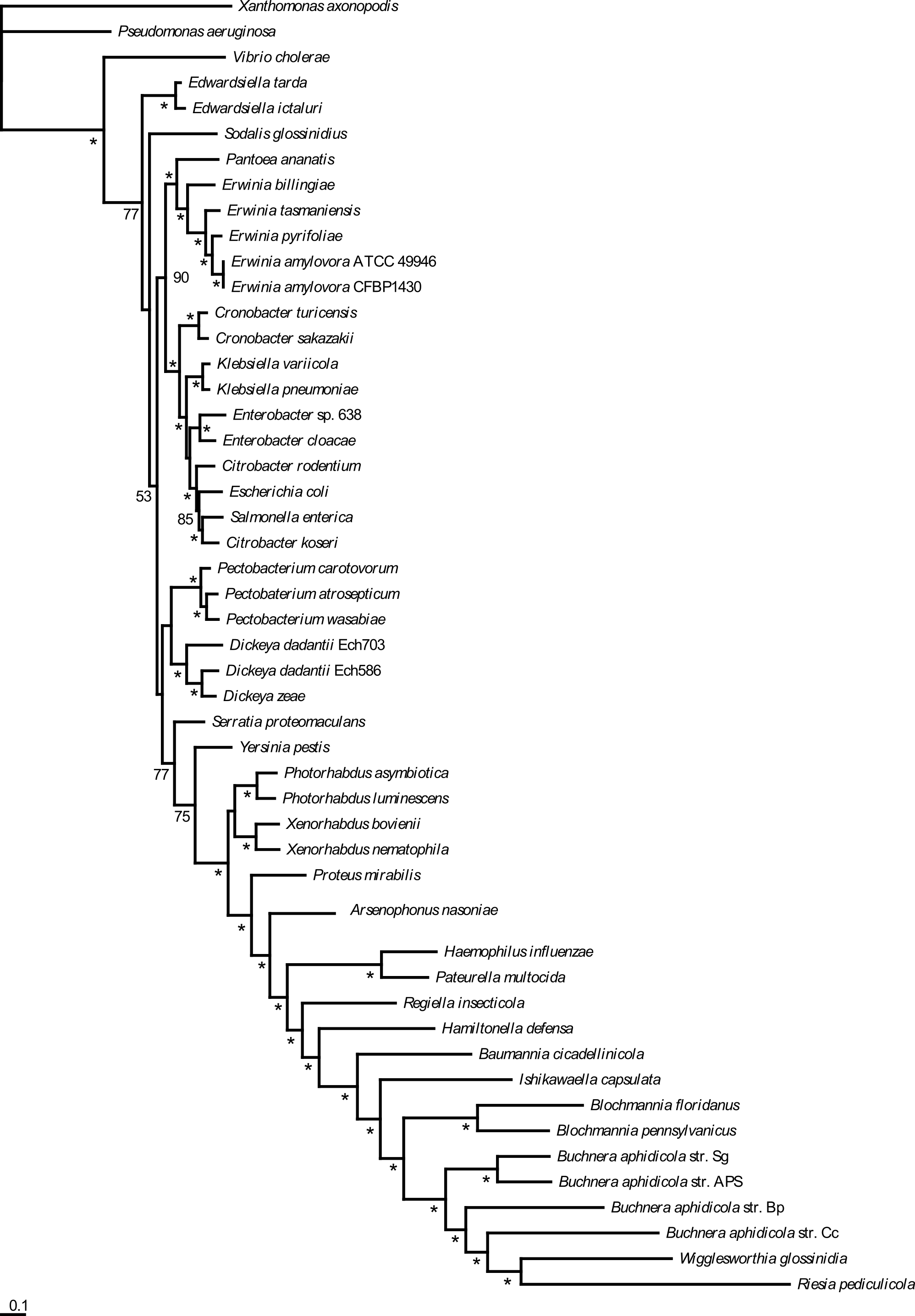
´

Additional file 2b - Phylogenetic tree inferred from the concatenated nuclotide matrix using ML under the GTR+I+Γ model. Asterisks represent nodes with bootstrap supports equal to 1.0.


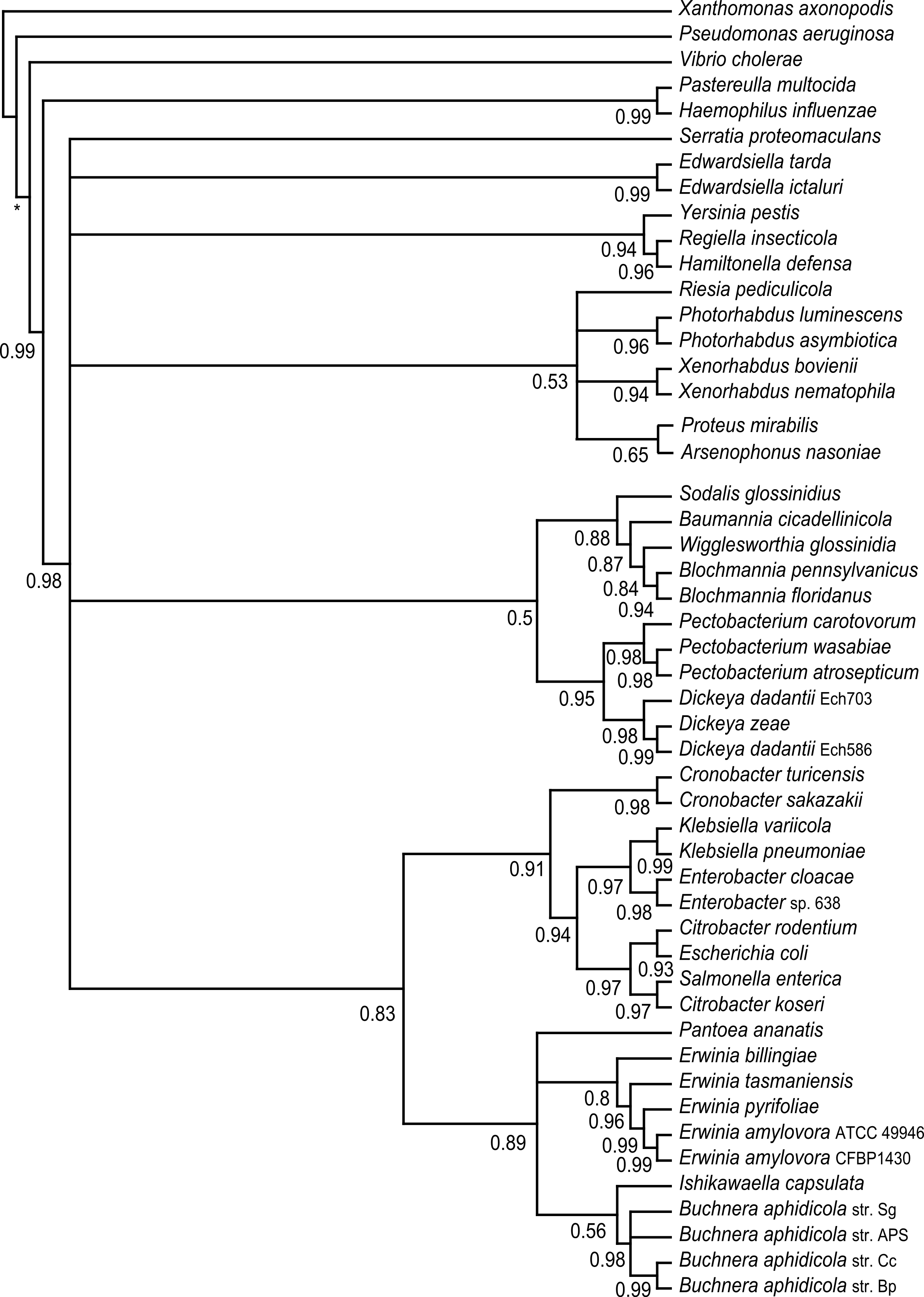


Additional file 2c - Cladogram derived from amino acid HP recoded matrix using PhyloBayes with the CAT model. Values at nodes represent posterior probabilities. Asterisks represent nodes with posterior probabilities equal to 1.0.


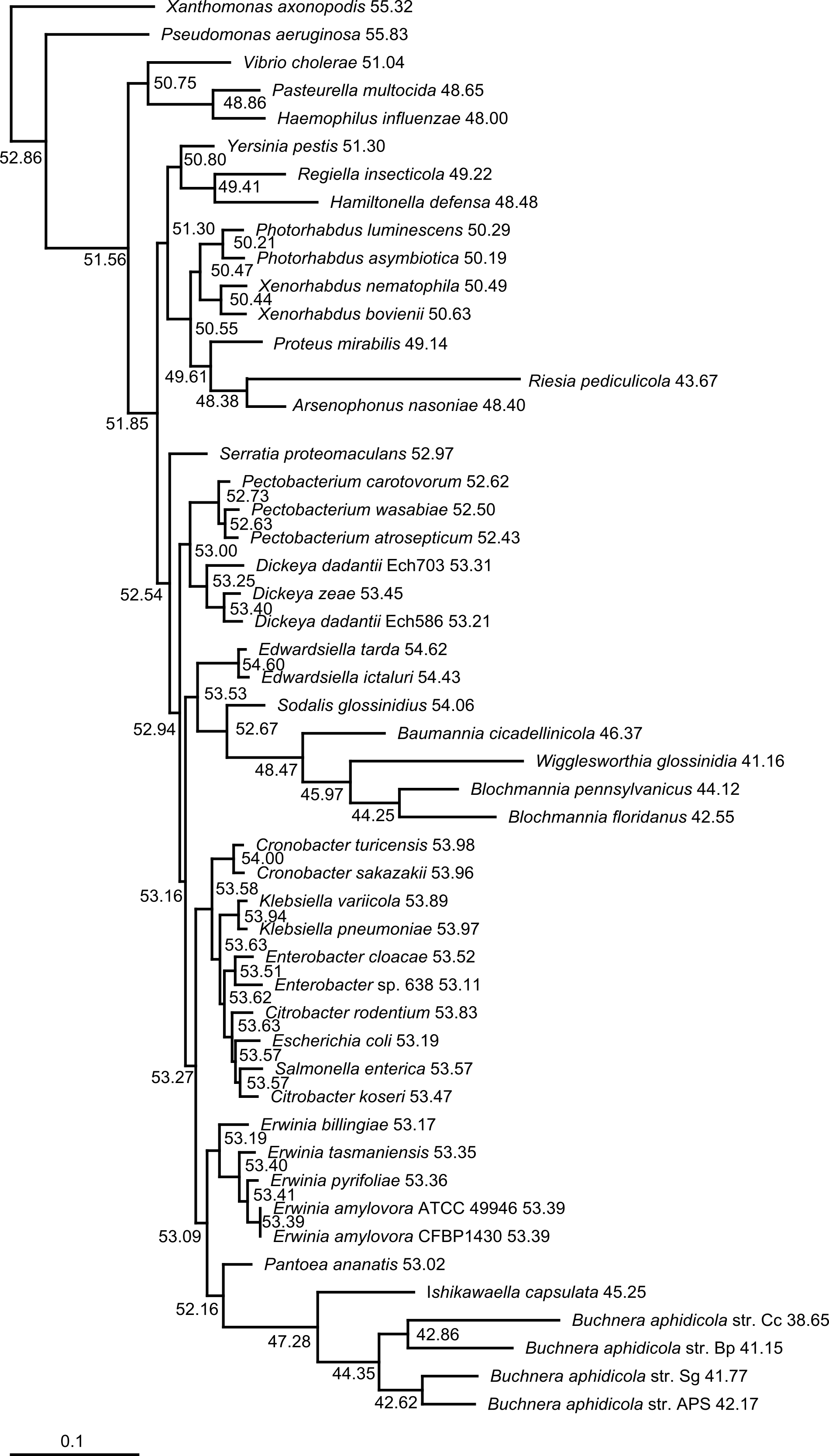


Additional file 2d - Phylogram inferred from the concatenated nucleotide matrix using nonhomogeneous model of evolution as implemented in nhPhyML. Polyphyly of insect symbionts was used as a starting tree (see 2n) for the analysis.Values at nodes and branches represent GC content.


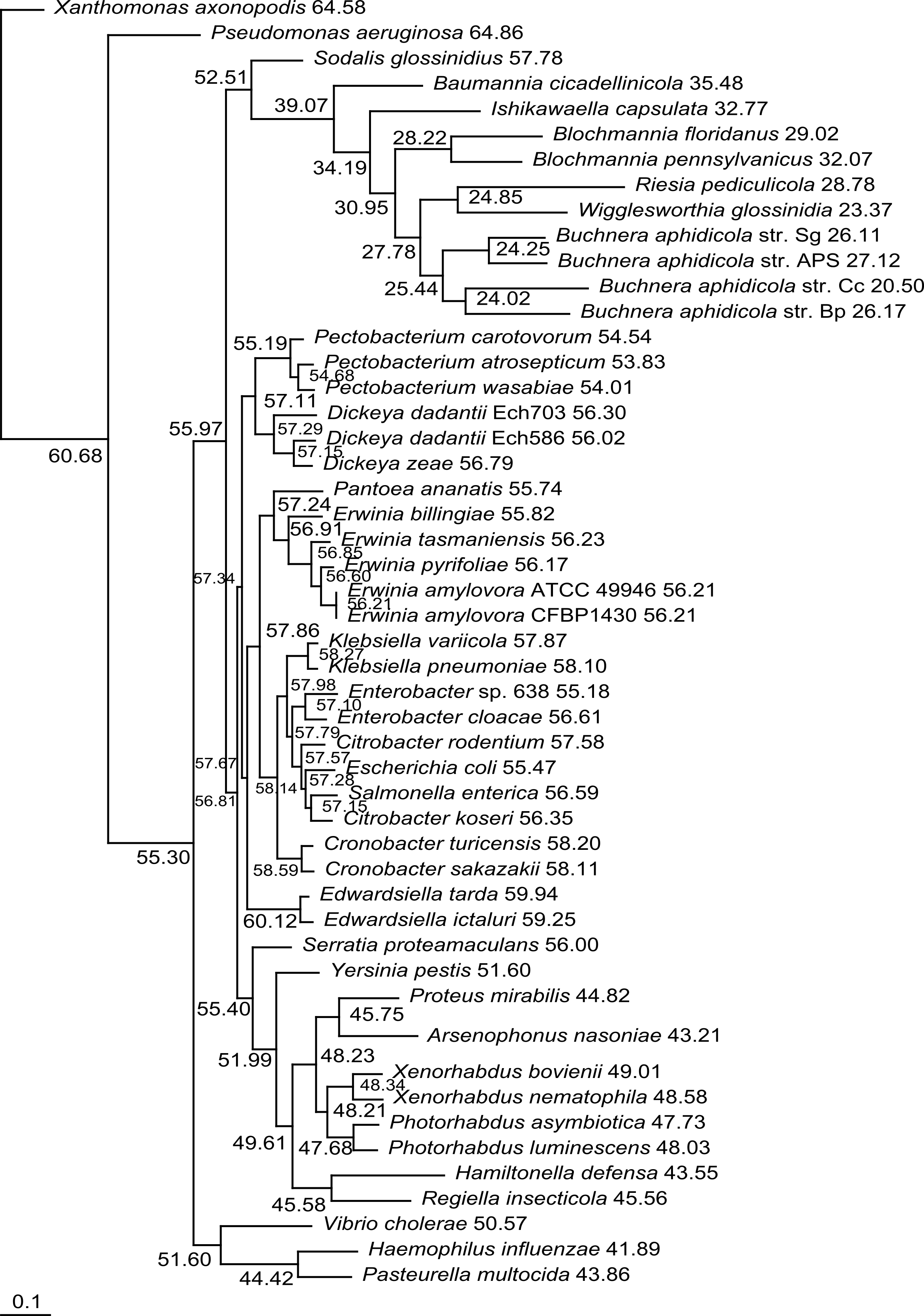


Additional file 2e - Phylogram inferred from the concatenated nucleotide matrix using nonhomogeneous model of evolution as implemented in nhPhyML. Monophyly of insect symbionts was used as a starting tree (2n) for the analysis.Values at nodes and branches represent GC content.


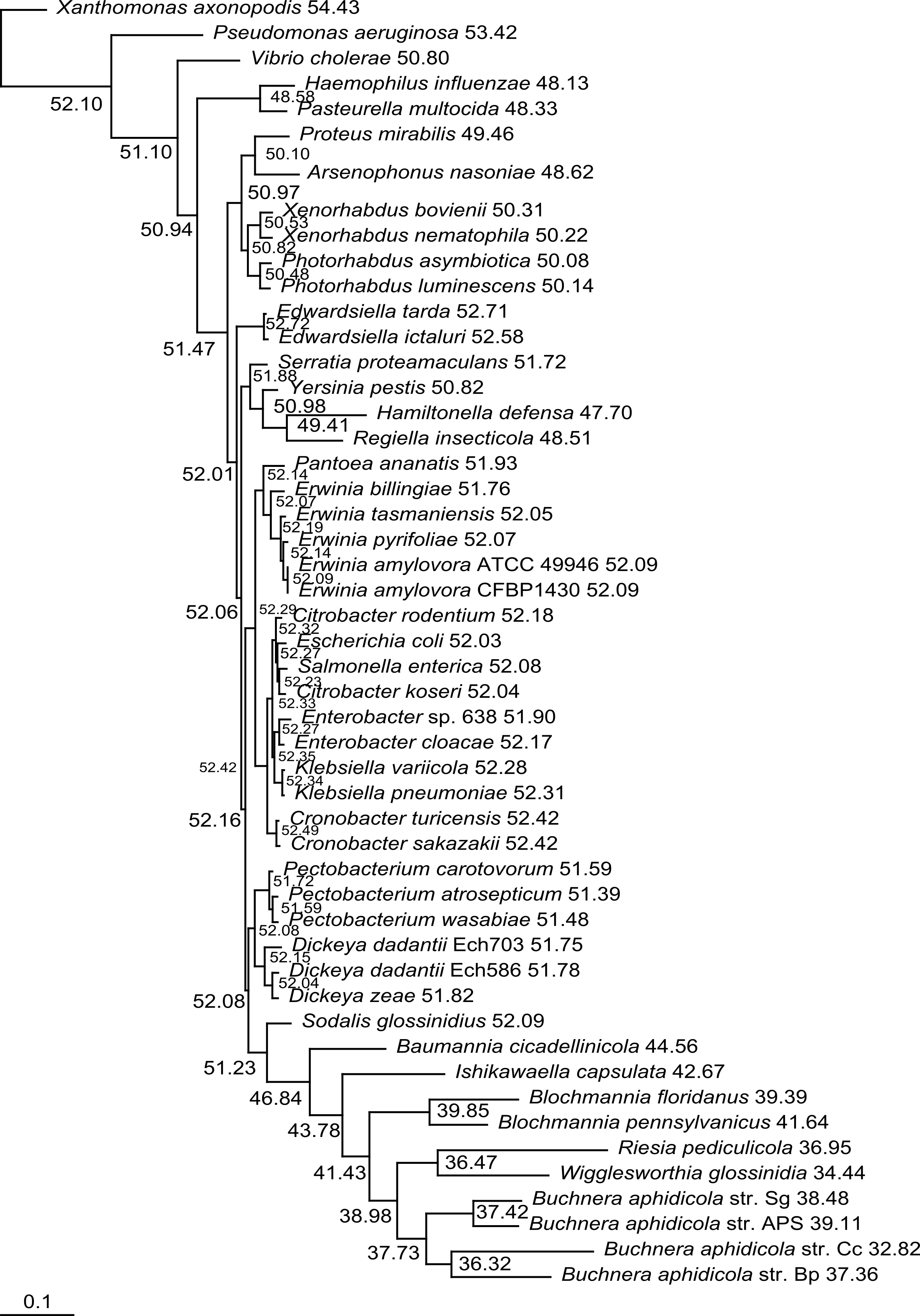


Additional file 2f - Phylogram inferred from the concatenated nucleotide matrix without third codon positions using the nonhomogeneous model of evolution as implemented in nhPhyML. Monophyly of insect symbionts was used as a starting tree (2n) for the analysis. Values at nodes and branches represent GC content.


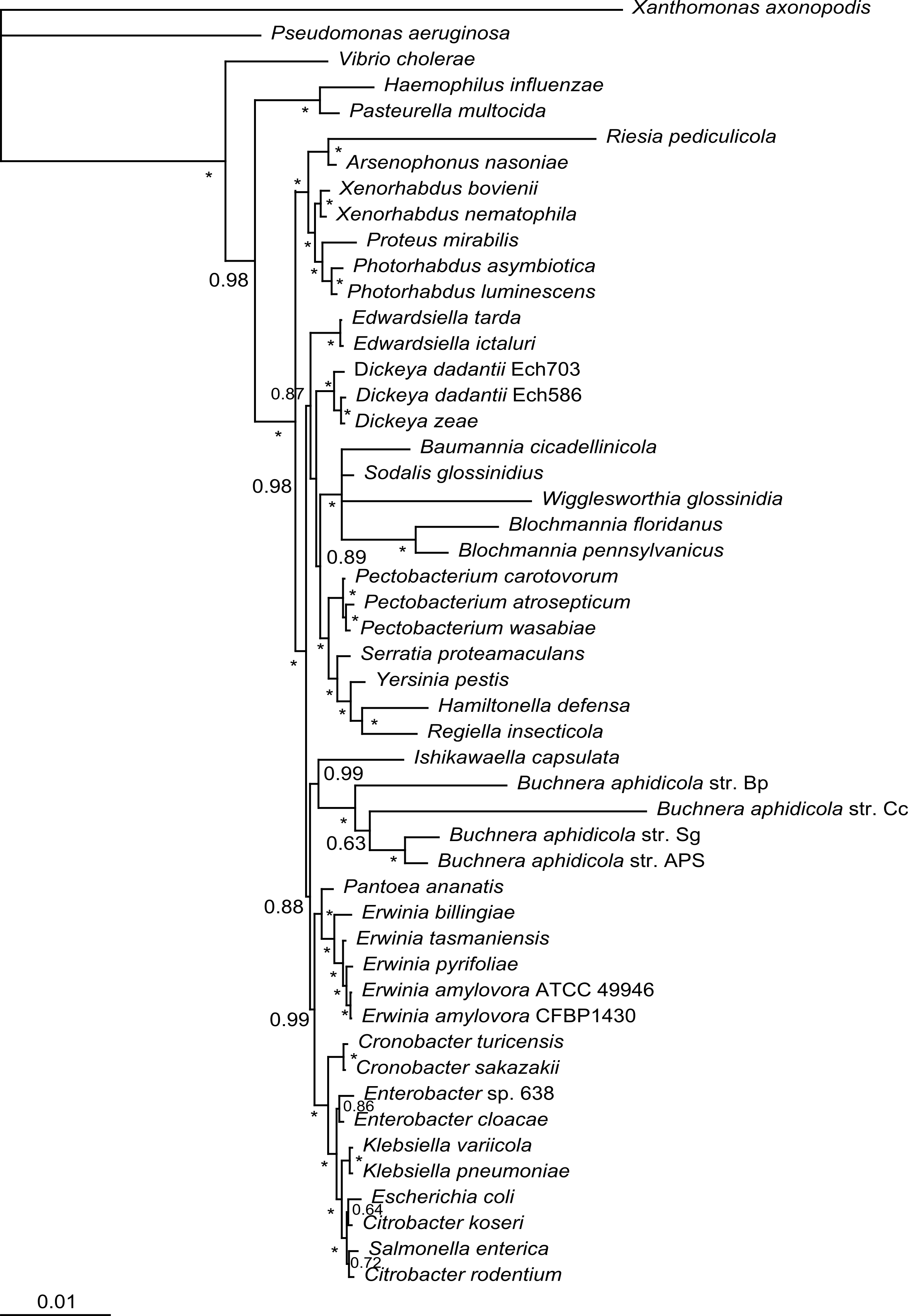


Additional file 2g – BI phylogram inferred from the concatenated nucleotide matrix with all AT/GC positions excluded (AT/GC 0). Values at nodes represent posterior probabilities, asterisks designate nodes with values equal to 1.0.


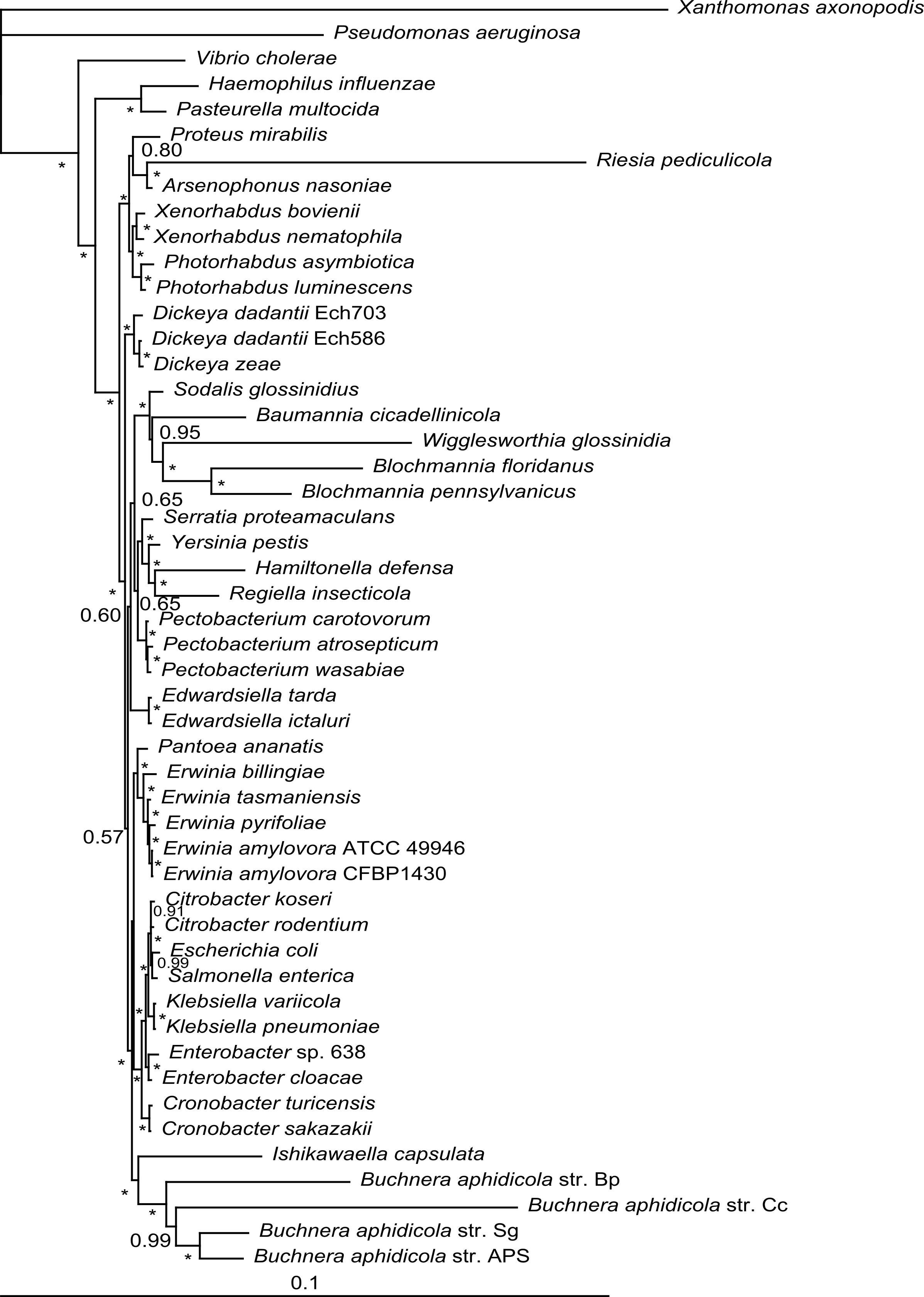


Additional file 2h – BI phylogram inferred from the concatenated nucleotide matrix with AT/GC positions excluded, but allowing for one taxon exception (AT/GC 1). Values at nodes represent posterior probabilities, asterisks designate nodes with values equal to 1.0.


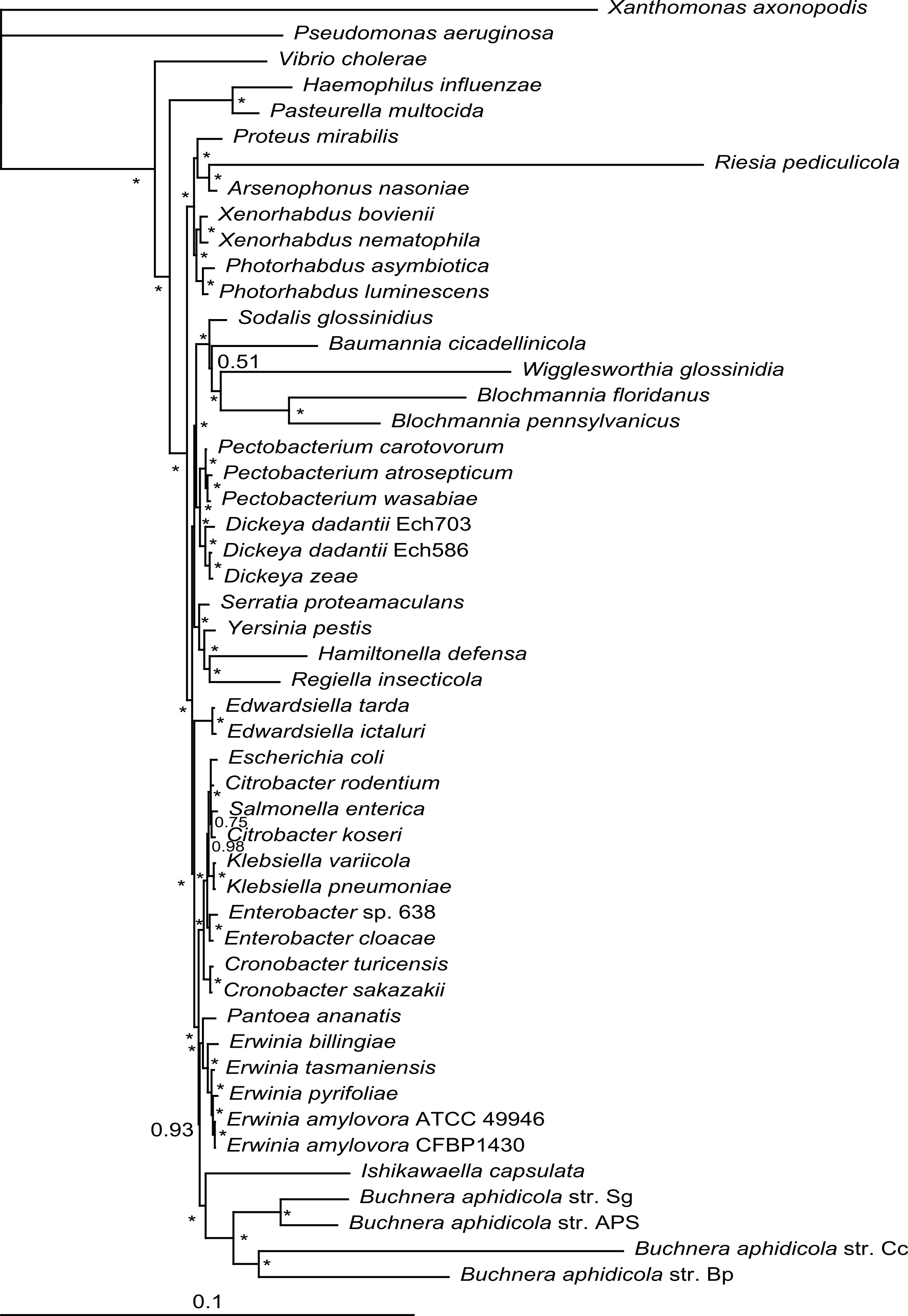


Additional file 2i – BI phylogram inferred from concatenated nucleotide matrix with AT/GC positions excluded, but allowing for two taxa exceptions (AT/GC 2). Values at nodes represent posterior probabilities, asterisks designate nodes with values equal to 1.0.


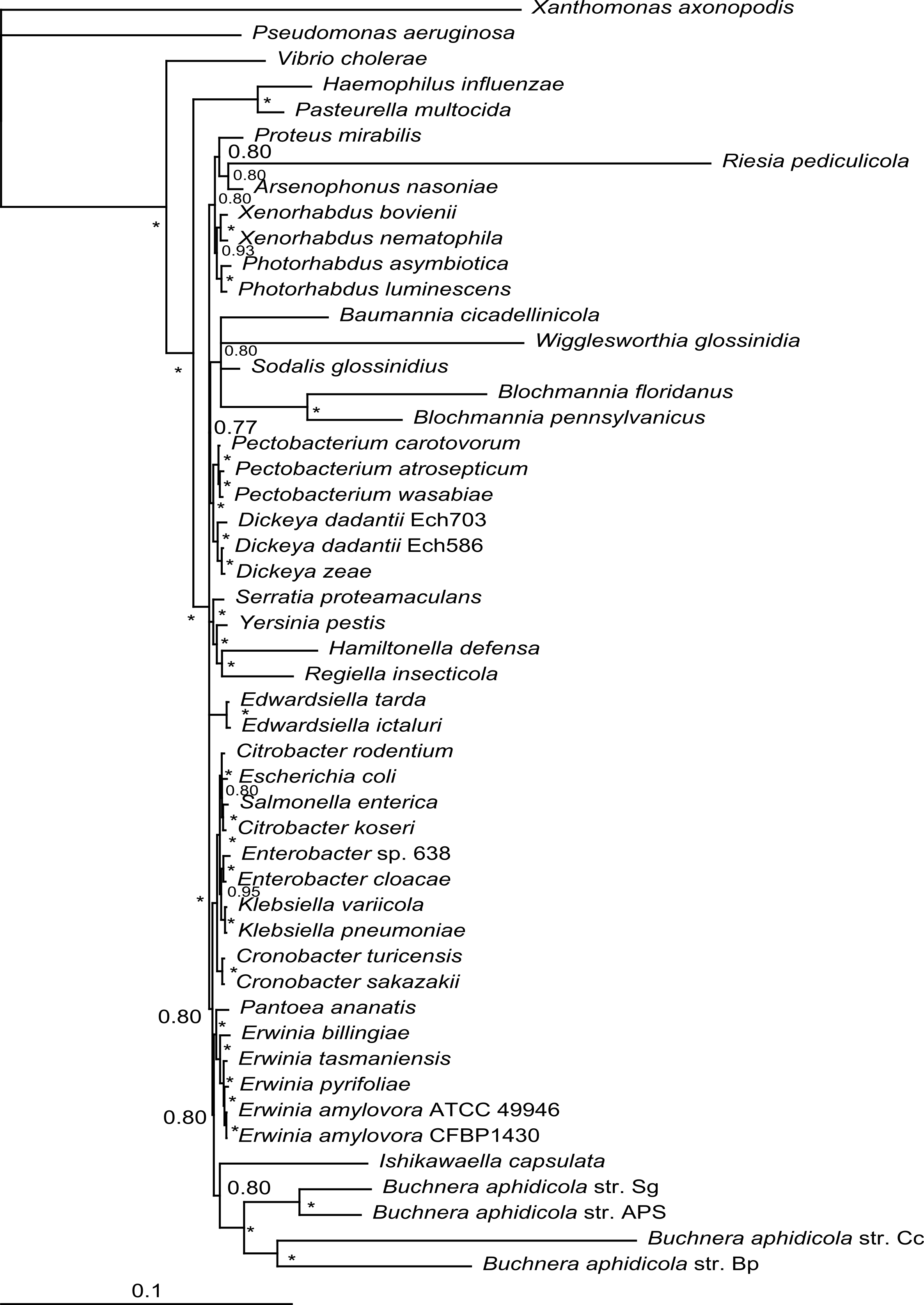


Additional file 2j – BI phylogram inferred from concatenated nucleotide matrix with AT/GC positions excluded, but allowing for tree taxa exceptions (AT/GC 3). Values at nodes represent posterior probabilities, asterisks designate nodes with values equal to 1.0.


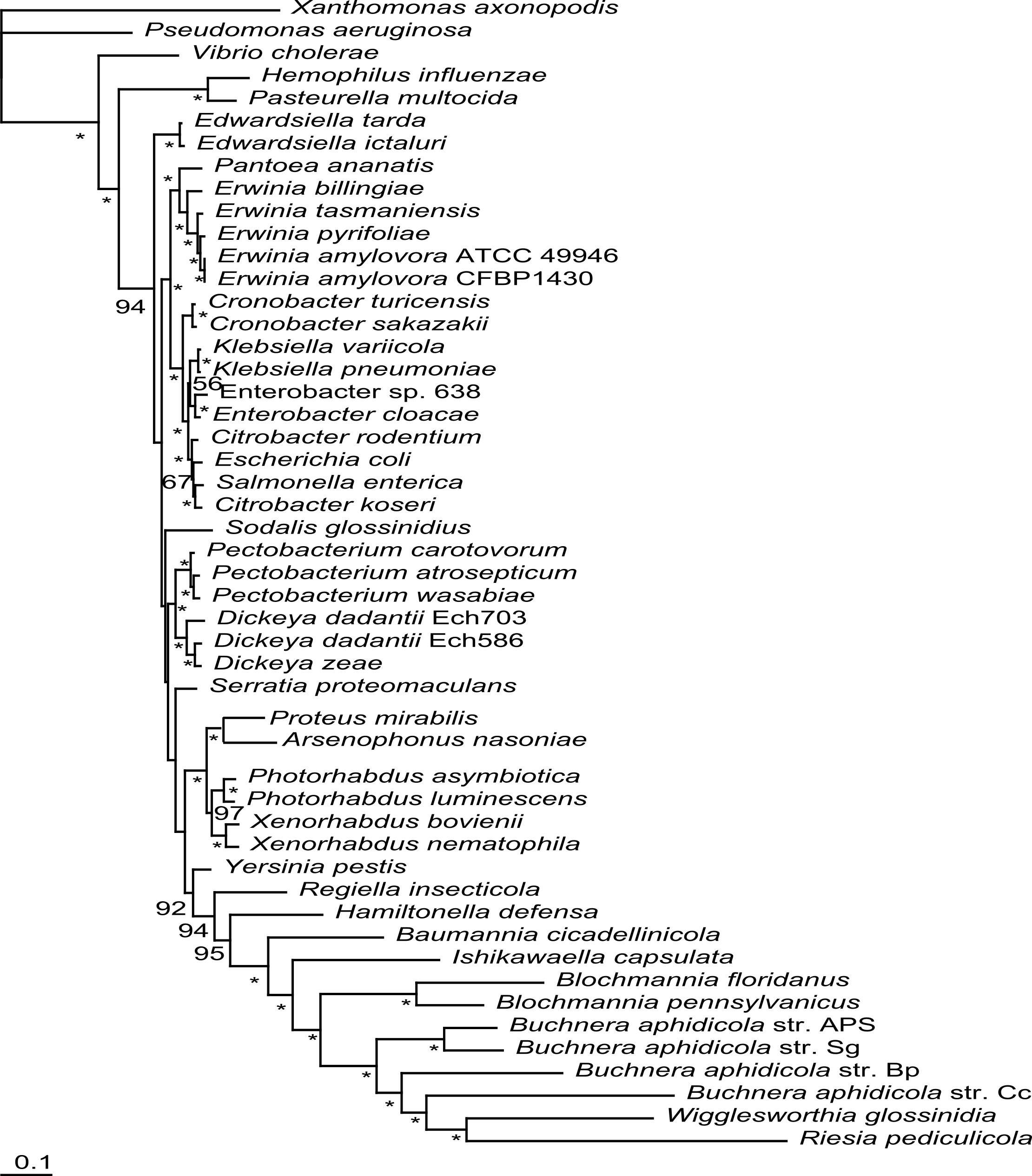


Additional file 2k - Phylogenetic tree inferred from the concatenated nuclotide matrix without third codon positions using ML under the GTR+I+Γ model. Asterisks represent nodes with bootstrap supports equal to 1.0.


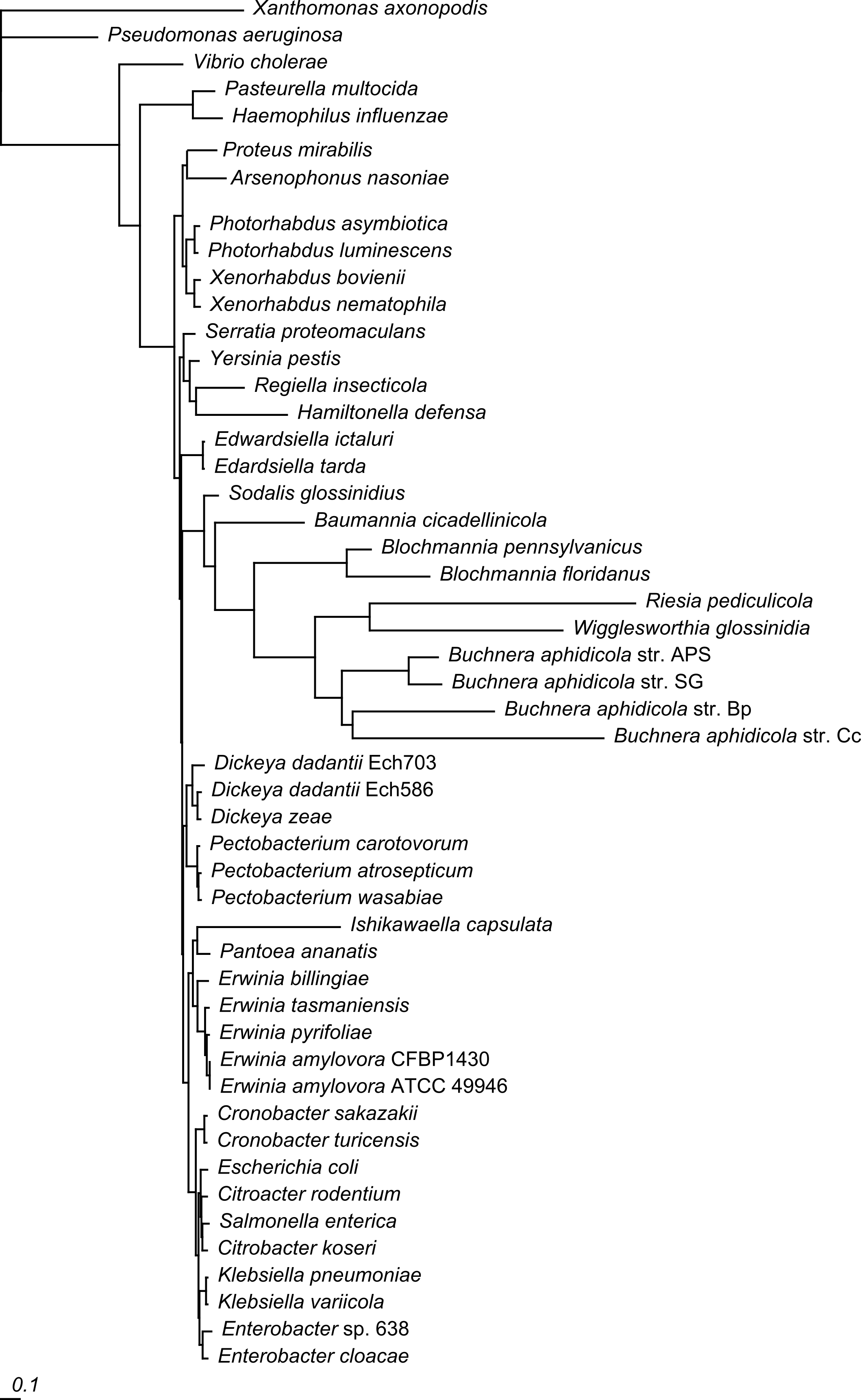


Additional file 2l - Phylogenetic tree inferred from the concatenated nuclotide RY recoded matrix using ML under the GTR+I+Γ model.


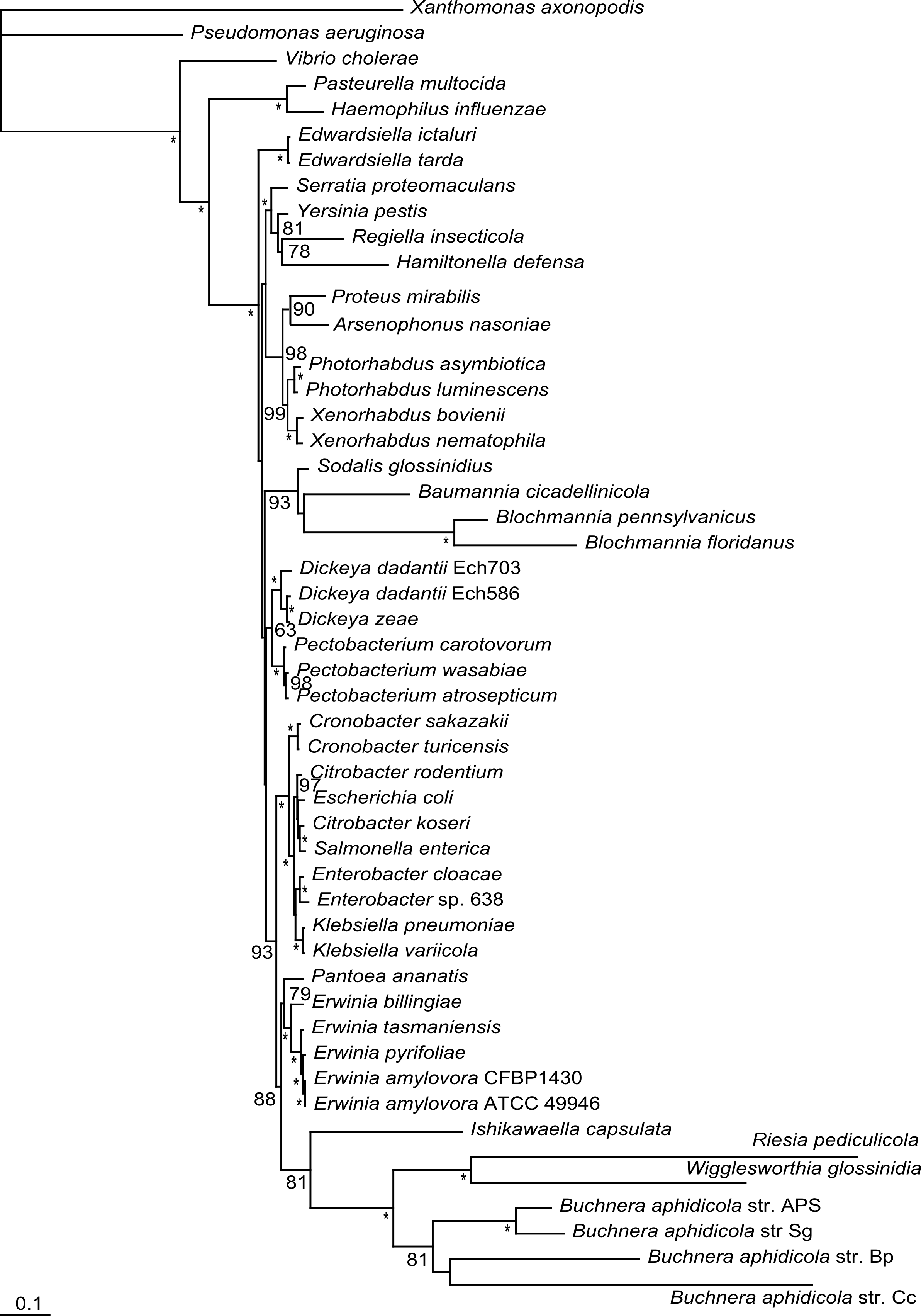


Additional file 2m - Phylogenetic tree inferred from the concatenated nuclotide RY recoded matrix without third codon positions using ML under the GTR+I+Γ model. Asterisks represent nodes with bootstrap supports equal to 1.0.

Additional file 2n - Two alternative topologies (polyphyly x monophyly of P-symbionts) used as starting trees for nhPhyML analyses.

Additional file 2o - Polytomic tree used to define monophyletic phyla (marked with black circles) for changes counting in slow-fast analysis.

Additional file 2p - Phylogram derived from 55-gene dataset using PhyloBayes with the CAT+GTR model. Values at nodes represent posterior probabilities. Asterisks represent nodes with posterior probabilities equal to 1.0.

Additional file 2q- Cladogram derived from amino acid dayhoff4 recoded matrix using PhyloBayes with the CAT+GTR model. Values at nodes represent posterior probabilities. Asterisks represent nodes with posterior probabilities equal to 1.0.

Additional file 2r- Cladogram derived from amino acid dayhoff4 recoded matrix using PhyloBayes with the CAT model. Values at nodes represent posterior probabilities. Asterisks represent nodes with posterior probabilities equal to 1.0.

Additional files 2s- Cladograms derived from amino acid dayhoff6 recoded matrices using PhyloBayes with the CAT+GTR model. Only one smybiotic lineage is retained and all other are excluded. Values at nodes represent posterior probabilities. Asterisks represent nodes with posterior probabilities equal to 1.0.
