# Supplementary material for: Multiple origins of endosymbiosis within the Enterobacteriaceae (γ-Proteobacteria): convergence of complex phylogenetic approaches
Source: BMC Biol. 2011 Dec 28;9:87. doi: 10.1186/1741-7007-9-87 (PMC3271043; doi:10.1186/1741-7007-9-87)
Supplement: Additional file 5 — Additional phylogenetic trees inferred from CAT and CAT+GTR unconverged chains. [file 1741-7007-9-87-S5.DOC]

Additional file 5 – Additional phylogenetic trees derives from unconverged PhyloBayes CAT and CAT+GTR analyses.

Additional file 5a - Phylogram derived from 14-gene dataset using PhyloBayes with the CAT model – the first chain. Values at nodes represent posterior probabilities. Asterisks represent nodes with posterior probabilities equal to 1.0.

Additional file 5b - Phylogram derived from 14-gene dataset using PhyloBayes with the CAT model – the second chain. Values at nodes represent posterior probabilities. Asterisks represent nodes with posterior probabilities equal to 1.0.

Additional file 5c - Phylogram derived from 55-gene dataset using PhyloBayes with the CAT model – the first chain. Values at nodes represent posterior probabilities. Asterisks represent nodes with posterior probabilities equal to 1.0.

Additional file 5d - Phylogram derived from 55-gene dataset using PhyloBayes with the CAT model – the second chain. Values at nodes represent posterior probabilities. Asterisks represent nodes with posterior probabilities equal to 1.0.

Additional file 5e - Phylogram derived from 69-gene dataset using PhyloBayes with the CAT model – the first chain. Values at nodes represent posterior probabilities. Asterisks represent nodes with posterior probabilities equal to 1.0.

Additional file 5f - Phylogram derived from 69-gene dataset using PhyloBayes with the CAT model – the second chain. Values at nodes represent posterior probabilities. Asterisks represent nodes with posterior probabilities equal to 1.0.

Additional file 5g - Phylogram derived from 69-gene dataset using PhyloBayes with the CAT+GTR model – the first chain. Values at nodes represent posterior probabilities. Asterisks represent nodes with posterior probabilities equal to 1.0.

Additional file 5h - Phylogram derived from 69-gene dataset using PhyloBayes with the CAT+GTR model – the second chain. Values at nodes represent posterior probabilities. Asterisks represent nodes with posterior probabilities equal to 1.0.
